# Touching Surfaces – Presence of microorganisms on antimicrobial metal surfaces on the International Space Station and in German schools

**DOI:** 10.1186/s12866-025-04316-6

**Published:** 2025-10-06

**Authors:** Carolin L. Krämer, Daniel W. Müller, Franca Arndt, Anna Rehm, Bernd Walkenfort, Aisha S. Ahmed, Aaron Haben, Alessa Schiele, Alina Auerhammer, Mike Hasenberg, Alessa L. Boschert, Ralf Kautenburger, Stefan Leuko, Stefan Janssen, Matthias Maurer, Frank Mücklich, Katharina Siems

**Affiliations:** 1 Department of Natural Sciences, University of Applied Sciences Bonn- Rhein- Sieg, Rheinbach, Germany; 2 Department of Applied Biology, Institute of Aerospace Medicine, German Aerospace Center, Cologne, Germany; 3 Department of Materials Science and Engineering, Chair of Functional Materials, Saarland University, Saarbrücken, Germany; 4 Institute for Medical Microbiology, Immunology and Hygiene, University Hospital of Cologne, Cologne, Germany; 5 Algorithmic Bioinformatics, Justus-Liebig University, Giessen, Germany; 6 Imaging Center Essen, Electron Microscopy Unit, Faculty of Medicine, University of Duisburg-Essen, Essen, Germany; 7 Department of Inorganic Solid State Chemistry, Elemental Analysis, Saarland University, Saarbrücken, Germany; 8 MVZ Laboratory Dr. Limbach & Colleagues eGbR, Heidelberg, Germany; 9 European Astronaut Center, European Space Agency, Cologne, Germany

**Keywords:** Antimicrobial surfaces, Aerospace microbiology, Space mission, Indoor microbiome, Confinement, ISS, Nanostructured surfaces, Surface functionalization

## Abstract

Microorganisms are an integral component of human health on Earth as well as for life on the International Space Station. However, inescapably, fomites in human habitats can serve as crucial niches for opportunistic pathogens. To explore potential countermeasures for the associated infection risk, the Touching Surfaces experiment evaluated antibacterial surfaces as high-touch surfaces on the International Space Station and on Earth. We used copper-based surfaces that integrate chemical antimicrobial properties with topography, thereby creating a metasurface. 16 S rRNA sequencing revealed that most bacteria found were human associated. While no significant distinction was observed between the microbial communities on the reference and antibacterial surfaces, isolation of microorganisms from the surfaces suggests that copper-based nanometer-structured surfaces exhibit enhanced antibacterial efficacy. The antibacterial efficacy of touched surfaces was reduced, as assessed by wet contact killing assays carried out using a methicillin-resistant *Staphylococcus aureus* (MRSA) isolate. The simplicity of implementing the surfaces allowed for straightforward testing of surfaces in both space and on Earth.

**Supplementary Information:**

The online version contains supplementary material available at 10.1186/s12866-025-04316-6.

## Introduction

Due to planned future crewed missions to deep space, human space habitat design and hygiene systems are particularly relevant topics [1, 2]. The microbiome of the built environment unveils a complex system which is present everywhere within our human-constructed environment. Beyond what meets the eye, our homes, schools, hospitals, and public spaces harbor a diverse community of microorganisms which influence our health and well-being [3, 4]. This becomes apparent during crewed space missions. The confined indoor living habitats combined with the diverse microbial communities carried by each individual astronaut, render microorganisms impossible to eliminate [5–7]. The International Space Station (ISS) represents a unique environment with its microbiome originating mainly from crew members as the ISS is separated from exterior contamination in between cargo resupplies and crew arrival and departure [5, 6, 8–10]. The isolation from external microbial sources in combination with the distinctive conditions in space such as microgravity and increased radiation, make the close monitoring of introduction and proliferation of potentially harmful organisms essential [5]. Microbial monitoring of the ISS has shown the persistence of microorganisms in filter systems, dust particles, as well as frequently touched surfaces including handrails, which astronauts use for moving through the space station [5, 6, 11]. Further characterization of individual strains has shown the presence of multidrug resistant microorganisms as well as opportunistic pathogens on board the ISS such as multi-resistant *Enterobacter bugandensis*, which is part of the *E. cloacae* cpx. which is an important multiresistant group, or *Acinetobacter pittii* [12–16]. Specifically, genes for antimicrobial resistance and adaptation to harsh environmental conditions as stress response have been investigated in detail [12, 17–20]. Some microorganisms can not only pose a threat to the crew’s health but also to the integrity of the spacecraft itself [21, 22].

Surfaces can be key niches for pathogenic microorganisms and opportunistic pathogens [23]. According to previous calculations, humans dispose up to 10 million bacterial and fungal cells per hour per person [24, 25], which are left behind not only in the air but also on frequently touched surfaces [26]. While the ISS itself is a unique habitat, its nature of being a built environment is transferable to other built environments such as the ones we have on Earth. Hence, research on antimicrobial surface concepts on the ISS can have a major impact also for life on Earth, for example in clinical settings with high-risk immunosuppressed patients or in remote and confined habitats, such as Antarctica, with limited medical support.

One strategy to reduce and control microbial contamination via surfaces is the use of antimicrobial active surfaces [27]. Copper surfaces have the potential to prevent the spread of pathogens due to their ability to rapidly eliminate pathogens upon surface contact [28]. Hereby, copper surfaces have demonstrated antimicrobial efficacy in various settings, including hospitals, and public transportation [29–31]. The antibacterial efficiency of copper surfaces was linked to the release of copper ions and a direct bacteria-surface contact [32, 33]. Additionally, differences in moisture content have been shown to impact the survival of copper-resistant *Escherichia coli* leading to lower survival in dry environments, while survival of copper-resistant *Enterococcus faecium* was higher in dry environments [34]. Hence, the antibacterial efficacy of copper-based surfaces is dependent on environmental influences such as humidity. However, even in dry environments, there is an aqueous phase surrounding the microorganism making antimicrobial activity possible [35].

Surface topography can be altered to increase antibacterial efficiency of the respective chemically antimicrobial surfaces such as brass or copper. Topographical and chemical surface functionalization by Ultrashort Pulsed Direct Laser Interference Patterning (USP-DLIP) has promising potential to improve antibacterial efficiency by modifying the surface at different periodicities [36]. These novel copper-based structured surfaces are already being tested using model organisms in the spaceflight experiment BIOFILMS [37]. In the “Touching Surfaces” project, novel copper-based structured surfaces were tested for their antimicrobial efficacy and application potential as frequently touched surfaces. For this purpose, nine different metal surfaces were implemented in specially designed hardware, so- called “Touch Arrays”. The Touch Arrays were tested on the ISS under space conditions and during a citizen science project in different schools around Germany. The latter also served as ground control. During the experiment, the surfaces were regularly touched by astronauts on board the ISS and by pupils from German schools. The implemented surfaces were then analyzed regarding their microbial contamination and surface properties.

## Materials and methods

### Touching Surfaces project

Touching Surfaces was part of the Cosmic Kiss mission of ESA astronaut Matthias Maurer, which took place from September 2021 to April 2022. The experiment was conducted over a time period of seven months (September 2021 to March 2022). For a more detailed description of the Touching Surfaces project see Krämer et al., [38] (in German language).

On the actively touched surfaces of the Touching Surfaces experiment, both the influence of topographical functionalization on biological transfer via fingerprint in general, as well as decontamination measures by utilizing antimicrobial active materials were investigated. Therefore, a specific setup of both material and topography of the touch surfaces has been considered: In the experiment, three topography modifications were tested: smooth, patterned close to bacterial scale (P ≈ Bac, 3 μm), patterned below bacterial scale (P < Bac, 800 nm). To determine the effect of micro- and nanometer scaled topography on both overall transfer and biofilm formation, stainless steel (AISI 304) as an inert non-antibacterial reference surface was selected. As antimicrobial active agents, copper (OF-Cu) and brass as a copper alloy (CuZn37) were chosen providing either higher (copper) or medium (brass) decontamination capacities in relation to their individual copper-content Linked with an altered Cu-ion emission. To allow insertion into the Touch Array experimental hardware, 1 mm sheets of copper (Cu > 99,95%), brass (Cu 63%, Zn 37%) (*Wieland*,* Germany*) and AISI 304 stainless steel (*Brio*,* Germany*) were cut into single metal plates of 10 mm x 25 mm dimension. While the steel samples were already provided with polished surfaces, the Cu-based materials were mirror-polished on an automated TegraPol-21 system (*Struers*,* Germany*). In the polished state, the plates of each material either underwent further topographic surface functionalization via Ultrashort Pulsed Direct Laser Interference Patterning (USP-DLIP) or were used as the smooth surface topography within the Touch Arrays.

### Ultrashort pulsed direct laser interference patterning (USP-DLIP)

Topographic surface functionalization was realized by means of USP-DLIP [39]. This technique enables the fabrication of periodic surface patterns down to the sub-micrometer scale with high pattern qualities even on the thermally high-conductive materials copper and brass. In parallel, the alteration of surface chemistry during processing is kept on a low level [40], which is mandatory to retain the antimicrobial capacities of the Cu-based materials [32] and was already evaluated in previous investigations [41]. USP-DLIP is conducted using a Ti: Sapphire Spitfire laser source (*Spectra Physics*,* USA*) emitting ultrashort pulses of tp = 100 fs pulse duration (Full Width at Half Maximum) at a centred wavelength λ of 800 nm and 1 kHz pulse frequency. In the optical DLIP setup, the seed beam passes an aperture defining the working beam diameter, as well as a wave plate that adjusts the polarization angle of the laser beam perpendicular to the generated pattern orientation. The beam is further divided by a diffractive optical element (DOE), while a lens system causes the two coherent beams to overlap on the sample surface inducing an interference pattern modulating the distribution of the laser intensity. In case of the two-beam interference setup used in these experiments, the intensity pattern showed a one-dimensional sinusoidal distribution, which is ablating the material in the regions of the intensity maxima, where the material specific ablation threshold is surpassed.

Topographies in the size scale of single bacterial cells (P ≈ Bac) were achieved by designing surface patterns with a periodicity of 3 μm, as described in Müller et al., 2021 [41]. For pattern scales smaller than single bacterial cell size (P < Bac), the pattern periodicity was altered to 800 nm. Patterning was conducted by modifying the lasing parameters according to Müller et al., 2020 [39] to achieve comparable pattern morphology on each material. The laser processed samples were subsequently selected to undergo immersion etching in 3% citric acid in an ultrasonic bath to remove process-induced surface oxide and sub-structures [40].

### Hardware assembly and disinfection

**Fig. 1:**
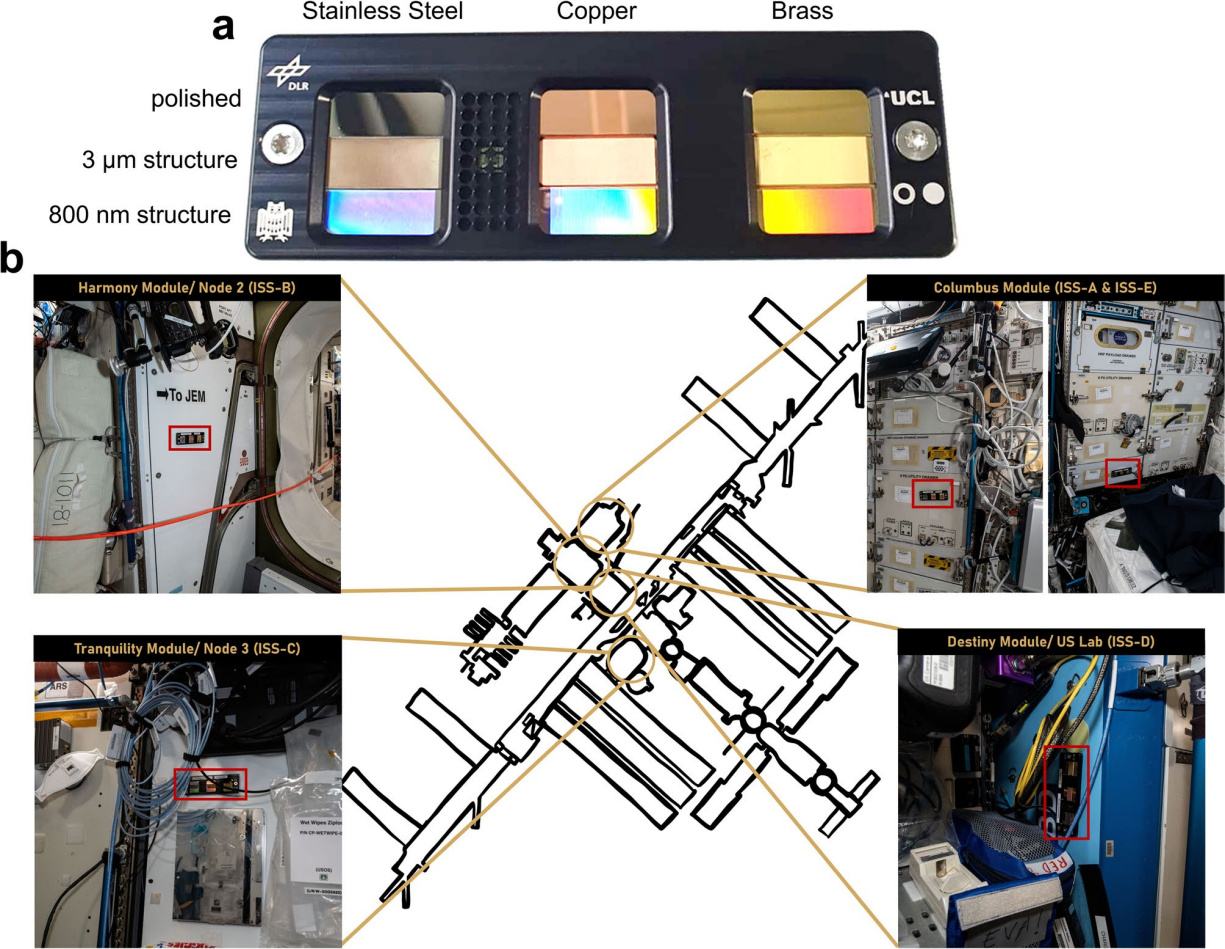
Set-up and Locations of Touch Arrays on ISS. **a** Sample surfaces were implemented into Touch Array Hardware as indicated in (**a**). The left slot was filled with stainless steel samples with a polished surface on top, a 3 μm structured surface in the middle, and an 800 nm structured surface below. The middle slot was filled with copper surfaces and the right slot was filled with brass surfaces with different topographies in the same order as described for stainless steel surface samples. **b** Two Touch Arrays were located in the Columbus module, and one Touch Array each was located in the Tranquility module/Node3, Destiny module/US Lab, and Harmony module/Node2. Shown in this figure are the Touch Arrays mounted onto the walls of the station in their respective module at the ISS. Pictures were taken by Matthias Maurer (© ESA)

Metal plates were placed in sterile 5 mL tubes and covered completely with absolute ethanol (> 99.8%) (*Carl Roth*, *Germany*). Absolute ethanol was used to minimize contact with water and thus corrosion of the copper-containing sample plates that would occur during autoclaving or treatment with 70% ethanol. After incubation of samples in absolute ethanol in an ultrasonic bath for 30 min (*Sonorex Digital 10P*,* Bandelin*,* Germany*), sample plates were taken out of the ethanol and left to dry under a laminar flow hood. Subsequently, sample plates were placed in a UV-C Lightbox for 1 min (total dose 840 J/m^2^) to ensure disinfection. Until further preparation, metal sample plates were placed in sterile petri dishes. The sample plates were then inserted in Touch Arrays, consisting of a top and bottom part, which are made up of black anodized aluminum (Fig. 1a). The hardware where the surfaces were implemented was designed and manufactured at the workshop of the German Aerospace Center. The Touch Arrays passed all tests for spaceflight.

For the assembly of the Touch Array, the case was disinfected using autoclaved cloths and sterile swabs, which were previously submerged with 70% ethanol. Subsequently, the metal sample plates were added to the bottom part, and for final assembly the top part of the Touch Array was added and fixed using two countersunk screws. Finally, the Touch Array was placed in a ziplock bag, which was previously disinfected using UV-C light (total dose 420 J/m^2^) irradiation for 30 min. Assembled, the Touch Arrays measured 8 mm x 136 mm x 45 mm each. An overview of the hardware with its implemented surfaces is given in Krämer et al., 2024 [38].

### ISS experimental setup

For the flight to the ISS, Touch Arrays were placed into individual insertion pockets inside a Nomex pouch, which was used for up- and download of the Touch Arrays. Two weeks after arrival on the ISS, five Touch Arrays were mounted using Velcro in different places, including contact areas, frequently and non- frequently used compartments. The Touch Arrays were mounted in the Columbus module (COL1F4 & COL1A4), Node2/Harmony module (NOD2P2), Node3/Tranquility module (NOD3A3) and the U.S. Lab/Destiny module (LAB1S6). For an overview of the location and subsequent analysis of the respective Touch Arrays refer to Table 1. Each Touch Array was touched at least 22 times and exposed to the environment on the ISS during the time of 22 weeks. The Touch Arrays were touched once a week, and each Touch Event was defined as the process of touching all five Touch Arrays onboard the ISS with the fingertip of the forefinger. All nine metallic test surfaces of each Touch Array were touched completely without a specific order. Prior to a Touch Event, hands were not cleaned for 1 h. Touch Arrays were not cleaned or included in routine cleaning activities of the station. The Touch Arrays were stored at room temperature and transportation from the ISS to the laboratories took 6 months.

**Table 1:** Overview of sampled Touch Arrays. For ISS and ground/school experiments five Touch Arrays were sampled each. For isolation and establishing of the experimental workflow for analyzing Touch Array samples, three additional Touch Arrays from schools were used. The respective location is given with the name, and subsequent analysis

Touch Array	Space/Earth	Location	Subsequent analysis
ISS-A	ISS	Columbus module	16 S rRNA Sequencing, Isolation, Antibacterial Efficacy (Contact Killing, ICP-QQQ)
ISS-B	ISS	Harmony module/Node 2	
ISS-C	ISS	Tranquility module/Node 3	
ISS-D	ISS	Destiny module/US Lab	
ISS-E	ISS	Columbus module	SEM (also on board the ISS via Mochii)
School-A	Germany	Saarland	16 S rRNA Sequencing, Isolation, Antibacterial Efficacy (Contact Killing, ICP-QQQ)
School-B	Germany	Bavaria	
School-C	Germany	Baden-Württemberg	
School-D	Germany	Bremen	
School-E	Germany	Saarland	SEM
School-F	Germany	Brandenburg	Isolation
School-G	Germany	Lower Saxony	
School-H	Germany	North-Rhine Westphalia	

### Ground experimental setup/citizen science project

As a ground control, eleven Touch Arrays were sent to schools throughout Germany, where they were frequently touched by students. The experimental setup was as described for the ISS. The overall amount of people touching the Arrays was higher for the ground control than for the flight experiment. For comparison of the microbial community and antibacterial efficiency of ISS Touch Arrays, four Touch Arrays from the schools were used. For comparison of SEM images of ISS Touch Array, one Touch Array from a school was used.

### Hardware disassembly and sample recovery

Upon return of the Touch Arrays to the laboratories, Touch Arrays were disassembled, and surfaces were retrieved under aseptic conditions. To analyze the microbial community, each surface was individually swabbed twice using sterile swabs, which were previously wetted with 40 µL 0.9% sterile NaCl (*Braun Melsungen AG*,* Germany*) solution. The first swab was used for subsequent DNA extraction, while the second swab was used for inoculation of cultivation media for subsequent isolation and identification of microorganisms. Surfaces were stored under aseptic conditions at room temperature until further analysis. As a control, blank swabs were taken which were wetted with 40 µL 0.9% sterile NaCl solution, and subsequently swirled through the air inside the laminar flow hood. Blank swabs were then processed in the same way as surface swabs during DNA extraction for microbial community analysis. Additionally, blank swabs were used for inoculation of media and incubation as a control for isolation of microorganisms.

### Microbial community analysis

For cultivation- independent microbial community analysis, swabs were placed in low bind tubes filled with 700 µL PCR- grade nuclease free water. DNA extraction was performed using ZymoBIOMICS DNA microprep kit (*Zymo Research*,* Germany*) kit according to the manufacturer’s manual with following modifications: After lysis of cells, supernatant was directly mixed with binding buffer and subsequently transferred to Zymo-Spin IC columns. DNA was eluted in 20 µL DNAse free water, which was preheated to 65 °C.

For subsequent phylogenetic analysis, sample preparation was performed using the Quick-16 S NGS Library Prep Kit (*Zymo Research*,* Germany*) according to the provided protocol with the following changes: 5 µL sample DNA was used. Normalization was performed using fluorescence standards. All samples were pooled and normalized with a quantity of 50 ng. Concentration was measured using QuBit (*Thermofisher Scientific*,* Germany*). For sequencing, samples were added with a final concentration of 10 pmol. Samples of blank swabs which were used as controls were sequenced twice each: Once normalized and once with a reduced concentration adjusted to sample concentration. Library denaturing and MiSeq sample loading was performed using the Illumina MiSeq Reagent Nano Kit v2 (500 cycles) (*Illumina*,* USA)* according to the manufacturer’s manual.

### Data processing

For basecalling of the obtained data from the MiSeq sequencing run, “bcl2fastq” (v2.19.0.316) was used and “cutadapt” (v2.10) [42] to generate trimmed FASTQ files. For quality control, “fastqc” and “multiqc” were used. After pre-processing the trimmed sequences were uploaded to the platform “Qiita” with the study ID 15175. Qiita was used for further processing including demultiplexing (“split libraries Fastq”), trimming to a length of 150 bp, and denoising was performed using “Deblur” (v2021.09) (https://qiita.ucsd.edu/; hosted at UC San Diego [43]). For computation of phylogenetic diversity distances, V1-V2 fragments were inserted into the reference phylogenetic tree of “Greengenes 22.10”. This was subsequently used for computation of weighted and unweighted UniFrac beta distances and Faith’s PD alpha diversity. Features were cut off at an abundance of < 10 over all samples, and sequences assigned as mitchondria and chloroplasts were filtered out as well. Additionally, we controlled for possible contaminations using decontam [44]. Rarefaction curves for all samples were computed via Faith’s PD. Jupyter notebooks for data analysis are made available in the additional material (https://github.com/jlab/microbiome_kraemer_touchingsurfaces). Computations were performed using the microbiome analysis pipeline QIIME2 (v2022_10) [45].

### Isolation and Identification of Microorganisms

After swabbing the respective surface with a swab which was pre-dampened with 40 µL 0.9% sterile NaCl, the swabs were added to 5 mL Brain Heart Infusion (BHI) (*Merck Millipore*,* Germany*) broth and incubated at 37 °C and 200 rpm for 48 h. After incubation, 10 µL each were transferred to sheep blood agar plates (SBA (sheep blood agar; Oxoid™, *Thermo Fisher.*,* USA*), Chocolate agar with vitox plates *(Thermo Fisher*,* USA*), extended spectrum beta-lactamase former agar (ESBL (chromID^®^ ESBL, *bioMérieux*,* France*)), and Columbia agar with 5% sheep blod and CNA (colistin + nalidixic acid) (*Bio-Rad*,* USA*) plates using an inoculation loop. Subsequently, pure cultures were obtained from each morphologically different colony. Isolates were identified by Matrix associated laser desorption/ionization- Time of Flight- Mass Spectrometry (MALDI-TOF-MS) (*Bruker Daltonic GmbH*,* USA*).

### Additional citizen science in schools

During the citizen science project of Touching Surfaces, additional isolation of microorganisms directly from agar plates was performed by the participating schools. Each school received 30 tryptone soy agar (TSA) plates, 30 mannitol salt agar plates, 30 R2A plates, 30 MacConkey agar plates, and 30 TSA contact plates (*Biomerieux*,* Germany*). The students were asked to touch the different agar plates with their fingertips. The TSA contact plates, which are petri dishes with raised agar, were used for sampling of frequently touched or highly trafficked surfaces around the school. The locations, which were sampled with the respective contact plates, were documented. The agar plates were sent back to the laboratories at the German Aerospace Center in Cologne. After incubation, colonies were transferred to selection agar plates for vancomycin-resistant *Enterococci* (chromID^®^ VRE, *bioMérieux*,* France*), MRSA (chromID^®^ MRSA, *bioMérieux*,* France)*, and ESBL (chromID^®^ ESBL, *bioMérieux*,* France*) for antibiotic resistance. Agar plates were then relocated to the University Hospital Cologne where they were incubated at 37 °C overnight. Isolation and identification were performed as described previously for isolates from Touch Arrays.

### Wet contact killing

To determine whether frequent touching and accompanying organic and microbial contamination have an effect on the antibacterial efficacy, wet contact killing assays were performed using an environmental MRSA isolate strain isolated from one of the participating schools. To create a defined surface area, a PVC ring with a defined inner diameter of 6 mm was added onto the surface. Untouched surfaces were disinfected using absolute ethanol (> 99.8%) and exposure to UV-C irradiation for 30 min. Touched surfaces were not cleaned prior to adding the PVC ring, but have been swabbed twice before for microbial community analysis. The environmental MRSA isolate strain was incubated on sheep blood agar (SBA) (*Merck*,* Germany)* plates overnight at 37 °C. To prepare a bacterial suspension, cell material of MRSA colonies was taken up with an inoculation loop and resuspended in 2 mL phosphate buffered saline (1xPBS) (*Carl Roth*,* Germany*) and the cell titer was determined using the McFarland standard. Cell titer was adjusted to a cell number of 10^6^ cells/mL.

For the contact killing assay itself, 40 µL of the bacterial suspension with a final cell titer of 10^6^ cells/mL was pipetted onto the defined surface area to create a monolayer of bacterial cells. The suspension was incubated on the surface for 10 min before taking it up again after pipetting up and down. The suspension was added to a sterile PCR tube for further testing. For determination of colony forming units, a 10-fold dilution series was performed from which 25 µL each were plated onto SBA and TSA plates.

### Determination of copper ion release

To analyze copper ion release, 5 µL of the respective samples from wet contact killing were added to 2995 µL of 0.69% nitric acid (HNO_3_) and analyzed using triple quadrupole inductively coupled plasma mass spectrometry (Agilent 8900 ICP-QQQ, *Agilent Technologies*, *Santa Clara*, *CA*, *USA*). A solution containing 10 mg/L each of Sc (1 g/L in 5% HNO_3_, *Alfa*^®^), Y (1 g/L in 2–3% HNO_3_, *Merck Certipur*^®^) and Ho (1 g/L in 2–3% HNO_3_, *Merck Certipur*^®^) in ultrapure water (0.055 µS/cm^2^; PURELAB^®^ Chorus 1, *Elga LabWater*,* High Wycombe*,* UK*) was prepared as an internal standard solution for all ICP-QQQ measurements. Argon 5.0 (Ar ≥ 99.999 mol%, ALPHAGAZ™ 1 Argon, Air Liquide) was used as plasma gas. For quantification purposes an external calibration was prepared, using Cu (1 g/L in 0.5 mol/L HNO_3_, *Merck Certipur*^®^) ICP-MS standard solution and the measurement (^63^Cu) was performed in He collision gas mode. To ensure that bacterial suspensions were inactivated before analysis using ICP-QQQ, for each sample, 20 µL of the mixture of 980 µL 0.69% HNO_3_ and 20 µL bacterial suspension, were plated on BHI agar plates, incubated for 48 h and evaluated for bacterial growth.

### Scanning electron microscopy

To investigate surface contamination, surfaces were analyzed using scanning electron microscopy (SEM). All plates from the Touch Array which had been installed on the ISS (ISS-E) received a 10 nm palladium sputter-coating, except for the 3 μm structured copper surface of ISS-E, which received a silver sputter-coating during a spaceflight experiment on the ISS.

To follow the IMCES routine protocols, sample plates of school Touch Arrays were chemically fixed using 4% formaldehyde and 2.5% glutaraldehyde in PBS for 15 min at room temperature (RT). Subsequently, surfaces were washed three times using de-ionized water before continuing with sample dehydration. For dehydration, samples were submerged in 30% ethanol and incubated for 10 min at RT before removal of the alcohol solution. This process was repeated for 50% ethanol, 80% ethanol, 96% ethanol and finally for three times 100% ethanol, dried on molecular sieves. Then, the surfaces were air-dried at room temperature for 30 min. Samples were mounted with adhesive carbon tabs onto 12.5 mm aluminum SEM specimen stubs (*Plano GmbH*,* Germany*).

Imaging was performed using a Zeiss Crossbeam 540 operating at a high tension of 3 kV with a beam current of 1 nA. To create overview images, mappings with a pixel size of 2.5 μm were made using the software package Atlas (v.5.3.2, *Fibics Inc.*,* Canada).* For in-depth analysis of the surface samples, images with 2048 × 1536 pixels and sizes of 18.61 nm, 3.72 nm, and 300 nm per pixel were created (image pixel size 1.49 nm) using the Zeiss acquisition software Smart SEM (*Carl Zeiss Microscopy GmbH*,* Germany*).

### Statistical analysis

Statistical tests for wet contact killing assays and ICP-QQQ measurements were performed using *Sigmaplot 14.5* (*Inpixon GmbH*,* Germany*). If the normality test (Shapiro-Wilk) was passed, an equal variance test (Brown-Forsythe) was performed. If the Brown-Forsythe method passed, and the differences in the mean values was greater than would be expected by chance, the Holm-Sidak method was performed as a pairwise multiple comparison procedure. If the equal variance test failed (*p* < 0.05), Tukey’s test was performed as a multiple comparison procedure. If the normality test (Shapiro Wilk) failed (*p* < 0.05) a Kruskal-Wallis one-way ANOVA on ranks was performed. To isolate groups which deviate from the others, Dunn’s test was performed as a multiple comparison procedure.

For further analysis of 16 S rRNA sequencing data we decided on a rarefaction depth of 1,500 reads per sample. Considering alpha diversities over different sampling depths, the chosen amount of reads supplied a balance between retaining samples and retaining diversity. For alpha diversity calculations, the metrics observed features, Shannon index and Faith’s phylogenetic diversity index were used. For beta diversity, the metrics weighted and unweighted UniFrac as well as Bray Curtis were utilized. Since metrics each show different aspects of diversity in a dataset, this variety was applied to showcase as many nuances in the composition of the data.

As statistical tests for imploring significance Mann-Whitney-Wilcoxon tests were used for alpha diversity and PERMANOVA tests with 999 permutations for beta diversity.

## Results

### Location of Touch Arrays inside ISS

Five Touch Arrays were located in different areas of the ISS (Fig. 1): Two Touch Arrays were located in ESA’s Columbus laboratory module, which houses experimental payloads such as Biolab and a microgravity science glovebox [46, 47]. Another Touch Array was placed in the Harmony module which connects the Columbus module to the rest of the station. The Harmony module is a living and working space for astronauts, connects international laboratory modules and allows cargo vehicles to arrive at its docking port. One Touch Array was located in another connection module, the tranquility module, which is home to exercise facilities, a bathroom, and life- support systems [48]. Another Touch Array was located in the destiny module, also known as U.S. lab as it carries the majority of U.S. payload, and was the first laboratory to be installed at the ISS [49]. As such, two Touch Arrays were located in connecting modules, and three Touch Arrays were located in a laboratory environment.

Table 1 provides an overview of the Touch Arrays’ locations and subsequent analysis. Due to the limited number of available samples, different analysis methods were used per Touch Array as stated in Table 1. Touch Arrays from all four locations were analyzed for microbial community analysis and antibacterial efficacy. One of the two Touch Arrays, which were located in the Columbus module was used for scanning electron microscopy (SEM) (Table 1: ISS-E), which included an additional analysis via the Mochii facility that was installed parallel to the Touching Surfaces experiment in 2021.

**Fig. 2:**
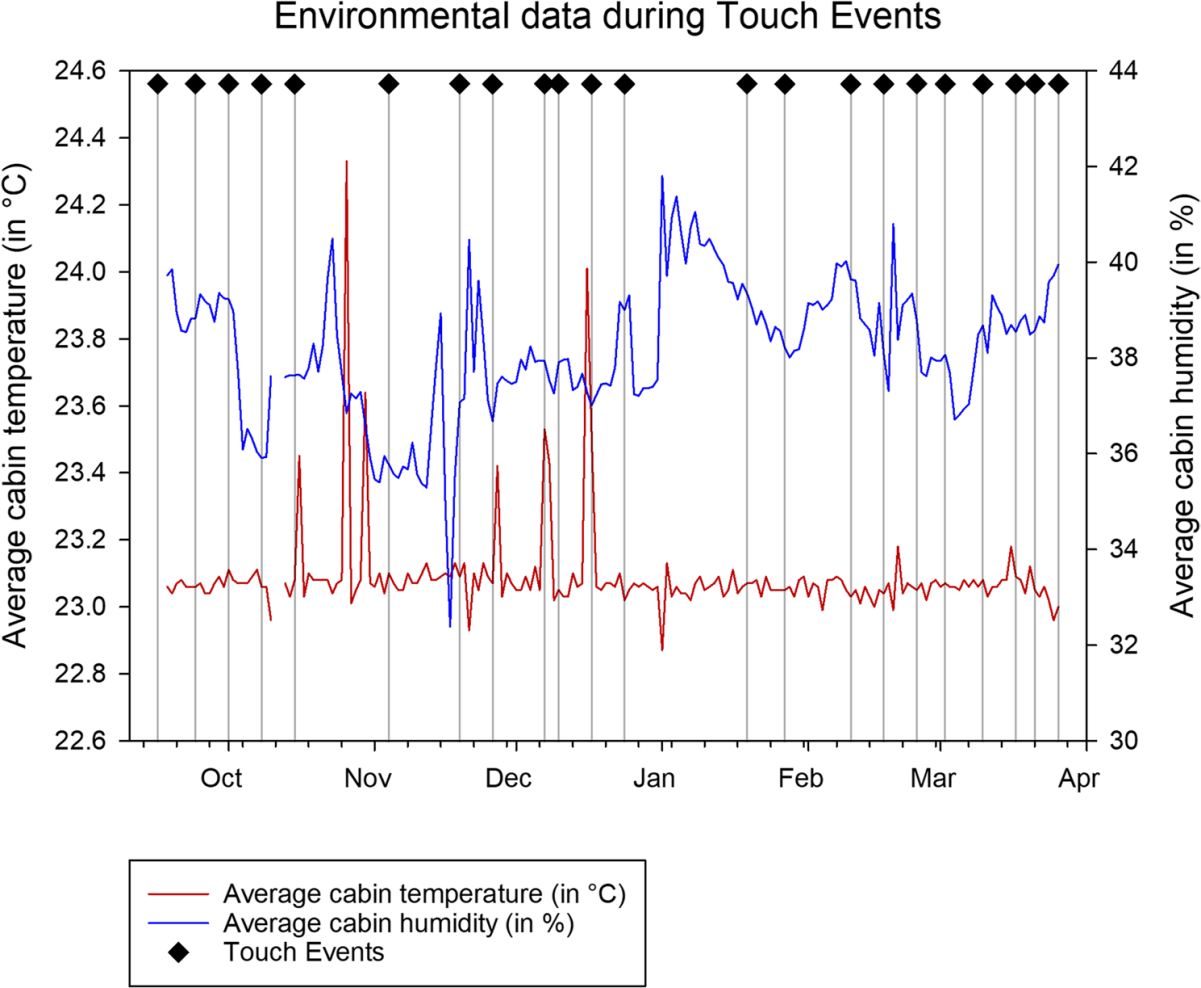
Environmental data during Touch Events on the ISS. The average cabin temperature and average cabin humidity is given and dates of Touch Events, where all Touch Arrays were touched, are marked. The average of cabin temperatures and cabin humidity is shown

The Touch Arrays on the ISS were touched a total of 22 times over 22 weeks. During the experiment duration and the stay on the ISS, the average temperature was 23.09 °C (± 0.14 °C) and the average Humidity across the different modules of the ISS was at 38.20% (± 1.41%) (Fig. 2).

### Citizen science project serves as ground experiment and is basis for isolation of microorganisms

As ground control for the Touching Surfaces project, students from schools in Germany were asked to take part by performing the experiment in the same way as astronauts on the ISS. The environmental data was documented daily (on school days) for three schools and for two schools at days of Touch Events using a thermo/hygrometer. The average temperature of the Touch Arrays in the schools was 21.3 °C (± 2.3 °C) and the average Humidity was 38.8% (± 9.3%) (Additional file 2: Additional Table 1). Exemplary Touch Events and an exemplary setup of one Touch Array in schools are shown in Additional file 1: Additional Fig. 1.

Citizen science is a collaborative approach to scientific investigation that involves the participation of citizens in the scientific process. It is an inclusive way of engaging and empowering people to contribute to scientific knowledge and understanding, while also helping researchers as citizens to contribute to data collection. It provides multiple benefits for the participants themselves, giving them the opportunity to learn about scientific concepts and contribute to research. Participation in the Touching Surfaces project was very well received by both students and teachers. The overall response was positive, and participants were eagerly waiting for results. Part of the citizen science project of Touching Surfaces involved the cultivation and isolation of environmental microorganisms with characterization focusing on multidrug-resistant microorganisms. Of over 500 agar plates sampled, one MRSA was isolated from an agar plate touched by students and used for further antimicrobial testing in this project.

### Surface structures remain intact despite putative organic contamination through frequent touching

**Fig. 3:**
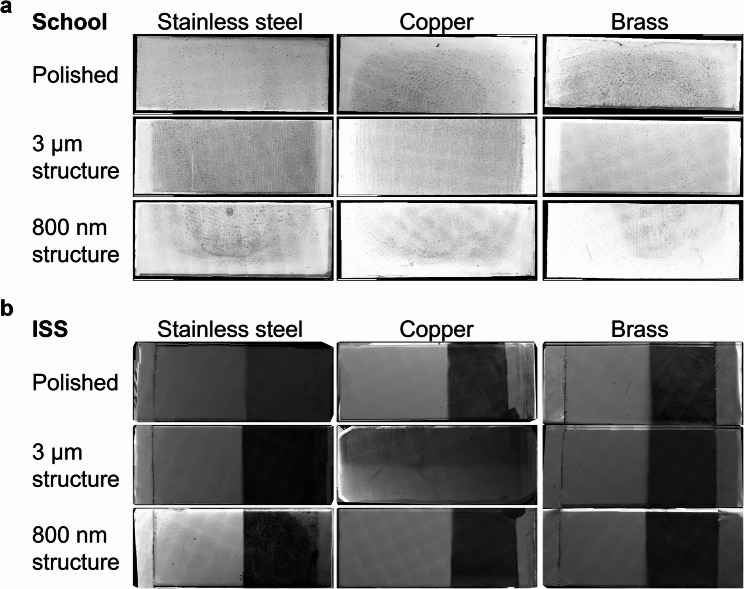
Mosaic images of surfaces from (**a**) school Touch Array and (**b**) ISS Touch Array. Using scanning electron microscopy, mosaic images of all Touch Array surfaces, which had been installed in a school environment (School-E) and in the Columbus module on the ISS (ISS-E), were acquired. After stitching the individual tile images, each resulting mosaic image shows the whole surface of one Touch Array with a dimension of 10 mm x 25 mm, respectively. To enhance visibility of the fingerprints, contrast and brightness were increased by 40% in the images shown in (**a**). The images in (**b**) appear in a “vertical split-view” which is a result of the sample treatment: To preserve the natural condition of a part of these precious samples, just half of each sample surface was sputter-coated with palladium while the other half was covered with tin foil during the sputtering process. The 3 μm structured copper surface was already analyzed on the ISS using the onboard scanning electron microscope (Mochii, Voxa, Seattle, USA). In this process this surface had been sputter-coated with silver which explains its different appearance in this figure

For investigation of robustness of surfaces as well as for deposition of possibly organic contamination, the sample plates of a Touch Array from a school (School-E) and of one Touch Array from the Columbus module (ISS-E) were analyzed using SEM. To visualize the entire Touch Array surface areas, SEM mosaic images were taken (Fig. 3). On steel surfaces of the school Touch Array (School-E), fingerprints were visible on structured surfaces but not on polished surfaces (Fig. 3A). Conversely, fingerprints were visible on all copper and brass surfaces of the school Touch Array (School-E), possibly due to oxidation of the copper present in these materials. In particular, organic debris from fingers left marks on these surfaces (Fig. 3a). Bacterial-like structures were found attached to the edges of the fingerprints along with what could presumably be organic debris.

On the surfaces of the ISS Touch Array (ISS-E), no fingerprints were detected. On the overview mappings of the surfaces, putative organic contamination is not clearly visible (Fig. 3b).

**Fig. 4:**
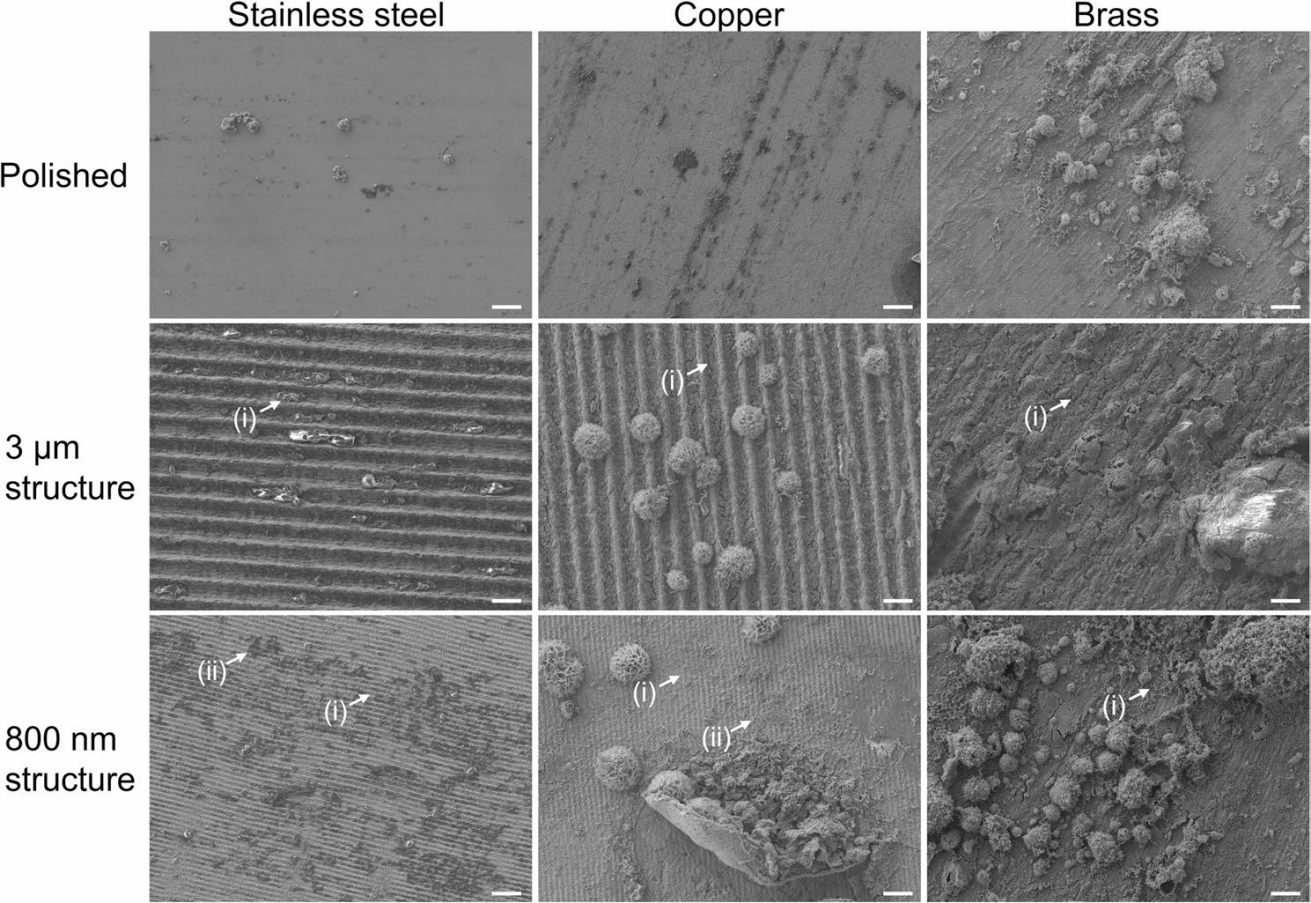
Exemplary scanning electron microscopy images of heavily contaminated areas of sample plates of school Touch Array (School-E) after frequent touching. Touched surfaces were analyzed using scanning electron microscopy. The upper legend indicates the respective metal, and the left legend describes the surface pattern of each surface sample. Sample surfaces had been installed in German school environment, where they were regularly touched by students. Sample surfaces were chemically fixed and imaged without previous sputter-coating. Pixel size of each picture is 27.91 nm. (Scale bar = 4 μm)

Whilst surfaces with 3 μm and 800 nm structures on all metals of the school Touch Array (School-E) remained largely intact despite frequent touching, putative organic contamination had accumulated in the grooves of the structures leading to aggregation of putative organic contamination (Fig. 4 (i)) including cocci-like structures (Additional file 1: Additional Fig. 4 (i), Additional file 1: Additional Fig. 6 (i)) and putative biofilm formation (Additional file 1: Additional Fig. 4 (ii), Additional file 1: Additional Fig. 6 (ii)) extending over the surface structures to form patches (Fig. 4 (ii), Additional file 1: Additional Figs. 4 and 6). Therefore, the antibacterial effect stemming from surface-structuring of copper-based metals may be reduced after frequent touching due to deposition of organic residues from the fingertips as well as from the environment.

**Fig. 5:**
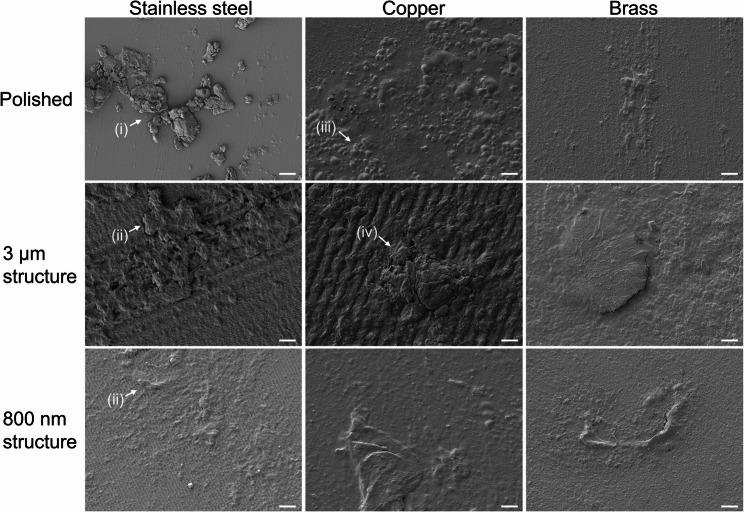
Exemplary images of heavily contaminated areas of sample plates from one Touch Array installed onboard the ISS (ISS-E). Touched surfaces were analyzed using scanning electron microscopy. The upper legend indicates the respective metal, and the left legend describes the surface pattern of each surface sample. The respective Touch Array had been installed inside the Columbus module on the ISS, where its surfaces were regularly touched by astronauts. The surfaces of the individual plates were not chemically fixed and for all plates, except the 3 μm structured copper surface, half of the surface was sputter-coated with palladium. For the 3 μm structured copper sample, the entire plate surface was already sputter-coated with silver onboard the ISS during a space experiment. Shown here are representative images of the non-sputter-coated part of each surface. Due to the sample pre-treatment on the ISS it was just possible to acquire images of silver-coated ultrastructure for the 3 μm structured copper plate. The pixel size of each picture is 27.91 nm, respectively. (Scale bar = 4 μm)

On polished stainless steel surfaces of the ISS Touch Array (ISS-E), another form of contamination was visible (Fig. 5 (i), Additional file 1: Additional Fig. 5 (i), Additional file 1: Additional Fig. 7 (i)). The structures here also appeared as organic mass such as mineral agglomerates, but no clear elements of microbial species were identifiable. Looking at both structured steel surfaces of ISS-E, putative organic contamination forms large patches over the 3 μm and 800 nm grooves, respectively (Fig. 5 (ii)). However, also in these images no putative microorganisms were spotted.

Equivalently, also on the three antibacterial-active copper surfaces of ISS-E, putative organic contamination was visible (Fig. 5, Additional file 1: Additional Figs. 5 and 7). On the polished copper surface, the image analysis revealed spherical structures with diameters of 0.5 μm up to several micrometers, potentially covered by a thin organic layer (Fig. 5 (iii). Additional file 1: Additional Fig. 5 (ii), Additional file 1: Additional Fig. 7 (ii)). Here, one could speculate that the spheres are cocci-shaped bacteria, or a fungal organism potentially organized as biofilm indicating potential production of extracellular polymeric substances. On the 3 μm structured copper surfaces, putative organic contamination was visible along the grooves with some of the putative organic contamination piling up to form a bulk over these grooves (Fig. 5 (iv)). On the 800 nm structured copper surfaces of ISS-E, the surface structure itself was still visible, but overlaid with organic layer reminiscent of a fluid smear, including rod-shaped structures (Additional file 1: Additional Fig. 5 (iii),Additional file 1: Additional Fig. 7 (iii)). On all three brass surfaces, individual surface topography was barely visible due to large thick patches of potentially organic surface contamination in the shown frames (Fig. 5, Additional file 1: Additional Figs. 5 and 7), including rod-shaped structures on the 800 nm brass surfaces (Additional file 1: Additional Fig. 7 (iv)). However, contamination of the surfaces was distributed unevenly, and pictures show heavily contaminated areas.

In summary, the electron microscopic investigation of Touch Array surfaces from both ground experiments in schools (Touch Array School-E, Fig. 4, Additional file 1: Additional Figs. 4 and 6) as well as from the ISS experiment (Touch Array ISS-E, Fig. 5, Additional file 1: Additional Figs. 5 and 7) revealed putative organic contamination on all surfaces while there was no such contamination detectable on untouched control surfaces (Additional file 1: Additional Figs. 2 and 3). Organic bulk contamination piled up along the 3 μm and 800 nm grooves, independent of the metal type. The majority of the laser-generated metal surface structures remained intact over the entire course of the touching experiments.

### Investigation of microbial communities using a cultivation-independent approach revealed the presence of many human-associated bacteria but did not determine differences in the microbial composition dependent on surface structures

**Fig. 6:**
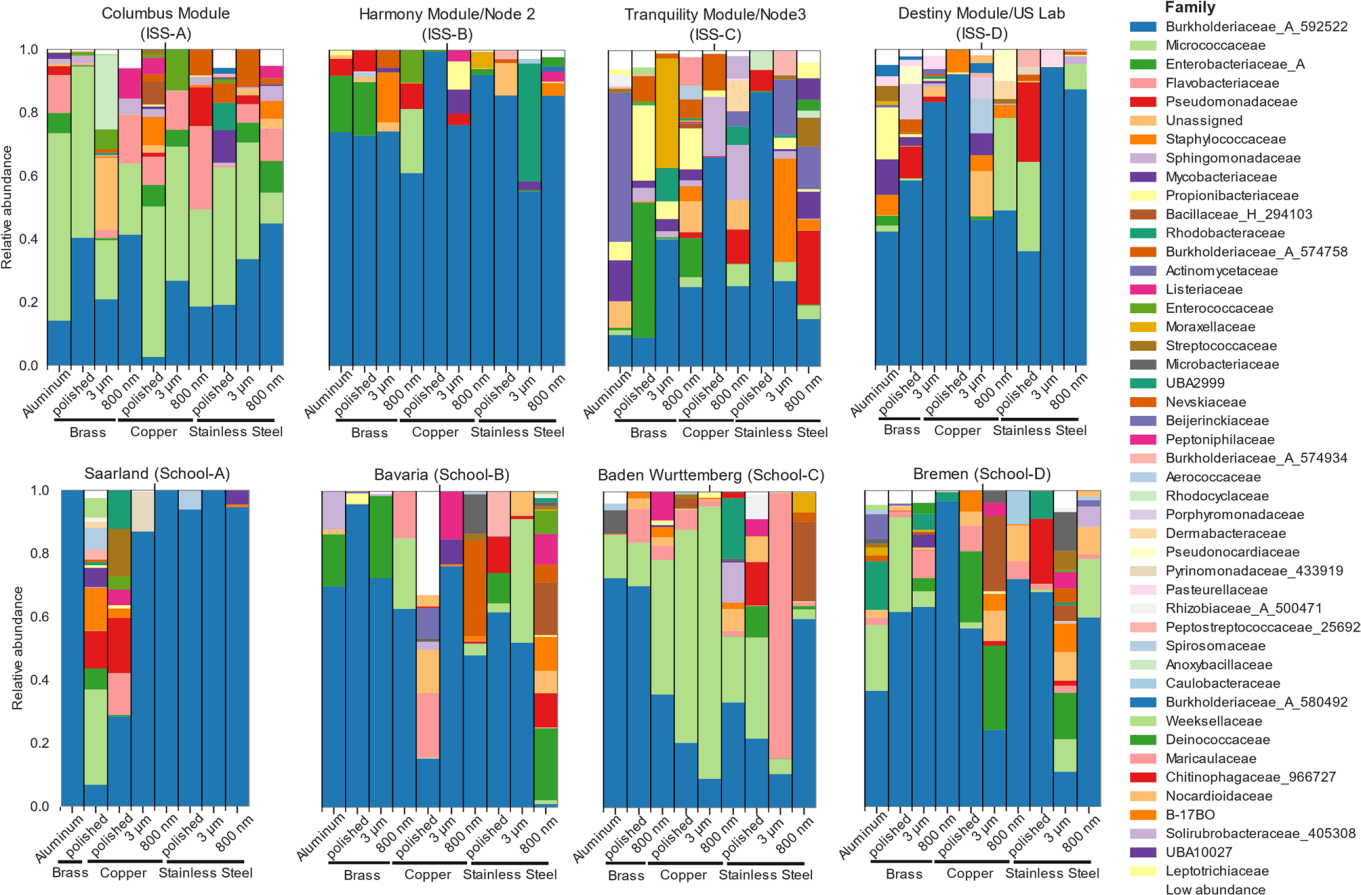
Bacterial communities on the different surfaces in respect to their location during the experiment. Taxa bar plots of decontaminated and rarefied data showing the relative abundance of detected bacteria using 16 S rRNA sequencing on family level

Overall, many of the detected bacteria using 16 S rRNA sequencing were human-associated such as *Micrococcaceae*,* Enterobacteriaceae*,* Pseudomonadaceae*, and *Staphylococcaceae* (Fig. 6). The most predominant family detected was *Burkholderiaceae.* Most relatively abundant on surfaces from the Touch Arrays on the ISS were *Burkholderiaceae*, *Micrococcaceae*,* Nevskiaceae*,* Propionibacteriaceae*,* Flavobacteriaceae*, *Pseudomonadaceae* and *Enterobacteriaceae.* The most predominant families on surfaces from school Touch Arrays were *Burkholderiaceae*,* Pseudomonadaceae*,* Micrococcaceae Enterobacteriaceae*,* Flavobacteriaceae*,* Marinilabacillaceae*, and *Bacillaceae*. To identify contaminants in the low biomass samples, we used blank swabs, taken while swabbing of the Touch Arrays in a laminar flow hood. The blank swabs were processed in the same manner as the sample swabs. During rarefication, 25% of the blank swab samples were lost. Sequencing of blank swabs showed that *Burkholderiaceae* were also relatively most abundant in blank swabs (Additional file 1: Additional Fig. 12).

**Fig. 7:**
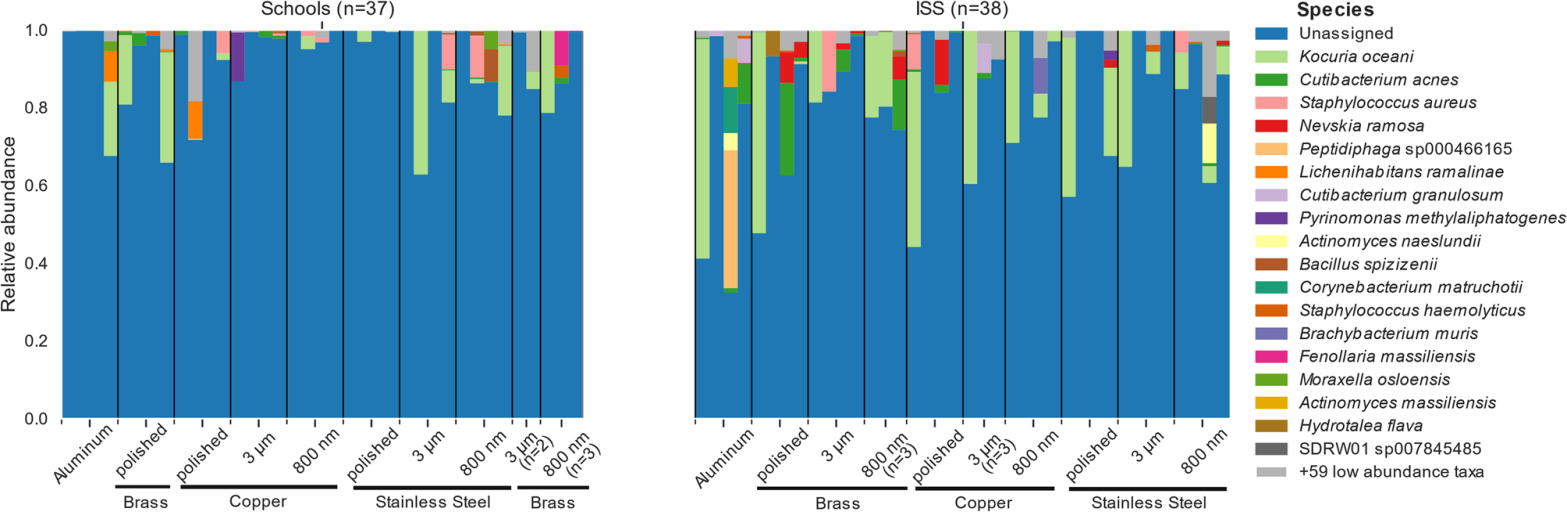
Relative abundance of bacteria on species level. Taxa bar plots for the detected bacteria on species level are given in respect to their location and the surface. After collapsing to species level 78 taxa were left, and after filtering out low abundant species 19 taxa remained. (*n* = 4 per sample group, if not stated otherwise in figure)

After collapsing the data to species level, 137 taxa were left. Looking at the species resolution, with the relatively highest abundance on Touch Arrays from ISS and schools, present were *Kocuria oceani*,* Cutibacterium acnes*,* Nevskia ramosa*,* S. aureus*, and *Staphylococcus haemolyticus* (Fig. 7).

We could not observe any statistically significant difference in the alpha- and beta- diversity of microbial communities on the different surfaces, i.e. surface structures, the metal itself or combination of surface structure and metal (Additional file 2: Additional Tables 5, 6, 7, 8, 9 and 10). Additionally, we could not detect statistical significance in the abundance of gram-positive, gram-negative, aerobic, facultative anaerobic, anaerobic, stress tolerant, potentially pathogenic, biofilm forming, or mobile elements containing bacteria in the microbial composition of the surface samples (Additional file 1: Additional Figs. 8-10). We also did not find statistically significant differences in the composition of the microbial communities on the surfaces of Touch Arrays in differently populated areas (Additional file 1: Additional Fig. 11).

However, 16 S rRNA sequencing reveals only genetic material present on the surfaces without indication of bacterial viability. Therefore, we also used a cultivation-dependent approach to detect bacteria that were still viable on the surfaces.

### Cultivation-dependent analysis suggests a potentially increased antimicrobial efficacy of copper-based surfaces with 800 nm structure

Cultivation and subsequent isolation of human-associated bacteria resulted in the isolates depicted in Table 2. The dominant microorganisms which were isolated from the ISS surfaces are mainly associated with the human microbiome, spore-formers, or have been previously isolated in confined environments such as the ISS [6, 50]. The isolation of many *Bacillus* species indicates that due to prolonged desiccation, selection was driven towards microorganisms which are desiccation-tolerant while different species might have been present on the surfaces but could not be isolated due to the long transportation time. The choice of medium and cultivation conditions focused especially on the cultivation of human-associated microorganisms. However, adding media to the swab possibly impacted the type of isolates as faster growing strains might have outcompeted slower growing strains. Additionally, due to antagonistic effects isolation of some species might have been favored, for example some *Bacillus* strains have been shown to produce antimicrobial compounds in co-cultures [51]. Additionally, surfaces were swabbed before for microbial community analysis through 16 S rRNA sequencing, which possibly reduced the number of recovered microorganisms as some might have already been removed. Most isolates were cultivated from steel surfaces, which have no chemical antibacterial activity and brass surfaces, which have moderate antibacterial activity, on Touch Arrays from both ISS and schools respectively. On the surfaces of the ISS Touch Arrays, no bacteria were isolated from any of the copper surfaces, regardless of the surface topography (polished, 3 μm, 800 nm). On the surfaces of the school Touch Arrays, single species were isolated from the polished and 3 μm patterned copper surfaces, but as with the ISS surfaces, none were isolated from the 800 nm patterned copper surfaces. In addition, no bacteria were isolated from the 800 nm brass surfaces on either the ISS or school Touch Arrays. *Micrococcus luteus* was isolated from the aluminum case of an ISS Touch Array itself.

**Table 2:** ISS and school isolates from Touch Arrays. Isolates, which were cultivated from Touch Array surfaces are listed dependent on their surface origin. From ISS Touch Arrays no isolates were cultivated from all copper surfaces and from 800 nm structured brass surfaces (no growth (n.g.)). From the aluminum case of the ISS Touch Arrays *Micrococcus luteus* was isolated. From school Touch Arrays no isolates were cultivated from 800 nm structured copper and brass surfaces respectively (no growth (n.g.))

ISS	Stainless Steel	Copper	Brass	Aluminum Case
Polished	*Bacillus cereus*	n.g.	*Staphylococcus haemolyticus*	*Micrococcus luteus*
	*Bacillus subtilis*		*Bacillus subtilis*	
3 μm structure	*Metabacillus niabensis*	n.g.	*Bacillus amyloliquefasciens* ssp. *plantarum*	
800 nm structure	*Bacillus cereus*	n.g.	n.g.	
	*Bacillus pumilus*			

School	Stainless Steel	Copper	Brass	Aluminum Case
Polished	*Bacillus subtilis*	*Micrococcus luteus*	*Alkalihalobacillus clausii*	*Kocuria marina*
		*Peribacillus simplex*		*Micrococcus luteus*
3 μm structure	*Aspergillus fumigatus*	*Bacillus subtilis*	*Bacillus flexus*	*Glutamicibacter creatinolyticus*
	*Micrococcus luteus*			*Alkalihalobacillus clausii*
	*Paenibacillus lautus*			*Pseudomonas oryzihabitans*
	*Paenibacillus polymyxa*			
800 nm structure	*Bacillus subtilis*	n.g.	n.g.	
	*Cytobacillus firmus*			
	*Staphylococcus epidermidis*			
	*Bacillus spp.*			
	*Cytobacillus firmus*			

Among the isolates from ISS Touch Arrays (Table 2), were besides *S. haemolyticus*, mainly spore-forming bacteria such as *Bacillus cereus*, *Bacillus subtilis*, *Bacillus pumilus*, *Bacillus amyloliquefasciens* and *Metabacillus niabensis*.

Among the isolates from school Touch Arrays (School-A, School-B, School-C, School-D), were *B subtilis*, *M. luteus*,* Pseudomonas oryzihabitans*,* Staphylococcus epidermidis* and *Aspergillus fumigatus* (Table 2).

### Frequent touching alters antibacterial efficacy

**Fig. 8:**
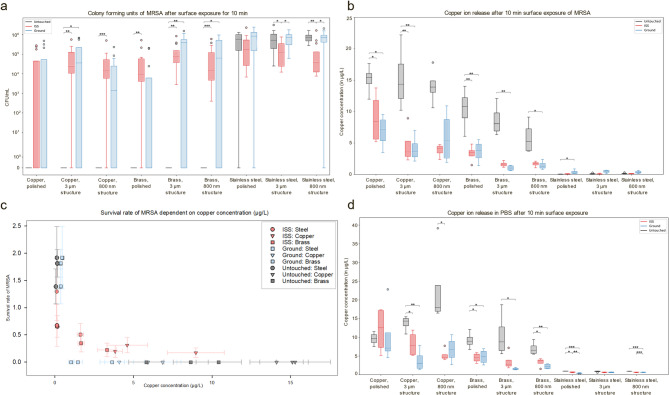
Effect of touching on antibacterial efficacy of surfaces. **a** Colony forming units of MRSA after 10 min surface contact. Colony forming units per mL (CFU/mL) per surface type are given for untouched and touched surfaces (ground and ISS) respectively. Four biological replicates with three technical replicates each are given per box. The y-axis is given as logarithmic. For determination of significance one-way ANOVA followed by a multiple comparison analysis was performed for each surface type between untouched, ISS and ground surfaces (Additional file 2: Additional Table 2). **b** Copper ion release in PBS after surface exposure of MRSA for 10 min. The copper concentration in µg/L per surface type is given for untouched and touched surfaces (ground and ISS) respectively. Four biological replicates are given per box. For determination of significance one-way ANOVA followed by a multiple comparison analysis was performed for each surface type between untouched, ISS and ground surfaces (Additional file 2: Additional Table 3). **c** Survival rate of MRSA dependent on copper concentration. The average survival rate of MRSA after surface contact for 10 min is given with the standard error of the mean on the y-axis. The survival rate was calculated by dividing the number of CFU/mL after contact killing by the initial CFU/mL (**d**) Copper ion release in PBS after surface exposure for 10 min. The copper concentration in sterile buffer in µg/L per surface type is given for untouched and touched surfaces (ground and ISS). Four replicates each are given per box. For determination of significance one-way ANOVA followed by a multiple comparison analysis was performed for each surface type between untouched, ISS and ground surfaces (Additional file 2: Additional Table 4). Significance level is given using stars (p < 0.05 = *), (p < 0.01 = *), (p < 0.001 = ***), if no significance is indicated mean values did not differ greater than would be expected by chance. Statistical significance testing is given in Additional Tables 2, 3 and 4

After the surfaces from both ISS and schools were swabbed, we additionally wanted to determine the antibacterial activity of the respective surfaces after frequent touching and swabbing. The antibacterial efficacy of copper-based surfaces is thought to be directly related to their release of copper ions [28, 41]. Therefore, the copper ion release in buffer from the touched and untouched surfaces was measured by triple quadrupole inductively coupled plasma mass spectrometry (ICP-QQQ) and compared within each sample surface category to determine the potential for antibacterial efficacy after frequent touching (Fig. 8d). While the copper concentration of the exposed stainless steel samples differed significantly from the unexposed sample plates (Additional file 2: Additional Table 4), this effect was neglected due to the overall low copper concentration, which was below the control concentration threshold. The copper ion release of touched and untouched polished copper surfaces did not differ significantly (Additional file 2: Additional Table 4). However, the copper ion release of untouched 3 μm structured copper surfaces was significantly higher than from both ISS and ground touched surfaces (Additional file 2: Additional Table 4), when compared to the copper concentration of touched 3 μm surfaces. Additionally, the copper ion release from untouched 800 nm structured copper surfaces differed significantly from touched surfaces on the ISS, with a decrease in copper ion release from the ISS surfaces (Additional file 2: Additional Table 4). Copper ion release from the moderately antibacterial brass surfaces decreased significantly for all surface structures between touched and untouched, except for the ISS brass surfaces with a 3 μm structure. The latter showed no significant difference (Additional file 2: Additional Table 4).

To further elucidate how the antibacterial efficacy is altered by frequent touching, touched surfaces were used for wet contact killing assays. An MRSA isolated from the citizen science project part of Touching Surfaces was used for the contact killing assays. When comparing colony forming units per mL (CFU/mL) after exposure to polished copper surfaces, MRSA did not survive on untouched copper surfaces after 10 min but survived on touched surfaces indicating a decrease in antibacterial activity after touching (Fig. 8a). This might be due to the formation of a passivation layer and the accumulation of putative organic contamination, that blocks copper ion release. However, survival on touched surfaces varied in between the groups (ISS, schools, untouched) tested of the different touched surfaces, which is possibly due to differential formation of a passivation layer and debris accumulation. No colony forming units of MRSA were detected after surface exposure to untouched brass surfaces after 10 min. However, MRSA survived on touched brass surfaces. The amount of CFU/mL detected differed significantly from the touched ISS copper surfaces, with an increase in survival (Additional file 2: Additional Table 2). Previous studies have shown that the release of copper ions into a suspension on surface contact also depends on whether bacteria are present or not and, if present, bacteria also influence the respective copper ion release [41]. Copper ion release from polished and 3 μm structured copper surfaces when exposed to MRSA decreased significantly after touching, indicating a decrease in antibacterial efficacy after frequent touching (Fig. 8b).

Apart from polished copper surfaces, all brass and copper surfaces showed a significant reduction of MRSA survival rate, correlating with a reduced copper ion release after surfaces were touched (Fig. 8c).

## Discussion

Following the theme of the Cosmic Kiss mission “Space for Life on Earth”, Touching Surfaces investigated antibacterial surfaces in direct application on the ISS, but also on Earth in schools. Thereby, we aimed at testing the impact of microgravity on the adhesion of microorganisms to surfaces, particularly antimicrobial surfaces, as well as the potential of these surfaces for application as high-touch surfaces.

Involving students from schools across Germany for the citizen science part, that simultaneously acted as ground control in Touching Surfaces, enabled public participation and raised interest not only in spaceflight, but also in microbiology, materials science and interdisciplinary projects. Hence, Touch Arrays used as ground control and reference were not placed in confined habitats, but instead in schools, which are more regularly frequented by a larger number of people. A more controlled setup in a confined habitat would have allowed a more direct comparison of microbial attachment between Earth and spaceflight focusing on the unique conditions on the ISS such as microgravity and increased radiation. Thus, the comparison of ISS Touch Arrays and school Touch Arrays compares the unique features of the ISS, including increased radiation, microgravity, and confinement, to build environment on Earth, that lack these distinctive features [52].

The results of the Touching Surfaces experiment from the ISS and from schools revealed that the surface structures themselves remained intact but putative organic contamination built up around the structures, particularly within the grooves, resulting in the formation of patches of possibly organic contamination. Consequently, the application of structured surfaces for everyday use and as frequent touch surfaces, needs to be further investigated and improved, for example by using specific cleaning agents [53].

Looking at the 16 S rRNA sequencing data, we did not see statistically significant differences in the composition of the bacterial communities depending on the different surface compositions, but we were able to identify statistically significant differences regarding the location of the Touch Arrays (ISS or school). Overall *Burkholderiaceae*, which encompass varied ecological roles such as insect symbionts, but also opportunistic pathogens in humans [54, 55], was the most abundant family in surface samples and blank samples. *Burkholderiaceae* are widespread in the environment including water, the human body, and surfaces, making them commonly detected in diverse microbial communities. Additionally, they have also been previously detected in microbiome data onboard the ISS [56]. Furthermore, some members of *Burkholderiaceae* such as *Cupriavidus metallidurans* have been shown to have a high metal tolerance [57]. Hence, the high relative abundance of *Burkholderiaceae* might be due to their persistence on metal surfaces or potential outgrow between surface exposure and sample collection/processing. On the ISS Touch Arrays, we found *Micrococcaceae*,* Enterobacteriacea*, and *Propionibacteriaceae* among the most prevalent taxa in the 16 S rRNA sequencing data. Skin-associated bacteria such as *Cutibacterium acnes* were detected indicating an influence of the skin microbiome on high-touch surfaces as has been previously described [8]. *Enterobacterales* were listed in the first priority group in the “priority pathogen” list of the World Health Organization with need for development of new antibiotics [15, 58].

An important point we considered during analysis was the quality of the sequencing data. The surfaces themselves were small (10 mm x 25 mm), and thus the biomass recovered was low. Nevertheless, the sequencing data passed quality control as described in materials and methods, but several difficulties must be considered during 16 S rRNA sequencing of low biomass samples as described in more detail in [59, 60]. Additionally, metal ions such as copper ions can also degrade nucleic acids, or inhibit cell lysis necessary for DNA extraction as well as polymerase activity [61, 62]. Lastly, the long shipment and storage time from the last Touch Event until analysis could have influenced results for example through blooming of microbial species which grow at room temperature [63]. However, the diversity of communities in soil, human gut and human skin samples appears to be unaffected by storage temperature after 14 days [64].

While no indications of changes in the microbial communities depending on the surface composition were found in 16 S rRNA data, we did find differences when cultivating and isolating bacteria from the respective surfaces. From copper-based surfaces (copper, brass) with 800 nm structure of the ISS Touch Arrays, no bacteria were isolated, pointing towards a trend of increased antibacterial efficacy of nano-structured surfaces in combination with antimicrobial effective metals. Generally, we did not isolate any bacteria from pure copper surfaces from the ISS Touch Arrays, which is indicative of the antimicrobial effect of copper as well as functionalized copper surfaces [28, 41]. No bacteria were isolated from the 800 nm copper and brass surfaces of the ground control Touch Arrays of the schools revealing a tendency towards an increased antibacterial efficacy. However, due to the surfaces being swabbed first for 16 S rRNA sequencing and then for isolation, microbial material might have already been removed through swabbing. Additionally, by adding the swabs to nutrient rich media, some species might have been favored before others.

The majority of the isolates belonged to the family of *Bacilli*, which are ubiquitous, common airborne contaminants and form spores to outlast unfavorable conditions such as desiccation [65]. Spore formation is a persistence strategy and provides protection against various conditions such as desiccation, UV- and ionizing radiation [66]. While *Bacillus* was among the most isolated bacteria from the surfaces, *Bacillus* was not among the most frequently relative abundant families present in 16 S rRNA sequencing data. There could be several reasons for this: Their dominance in isolates could be due to their ability to form spores enabling them to stay dormant for a longer period of time. Additionally, DNA extraction of spores can be more difficult due to the structure of spores. Research has shown that *Bacillus* species are common isolates from the ISS including a variety of species, such as *B. pumilus*, *B. subtilis*, and *B. amyloliquefaciens* [50]. While some of these species are known for their high resistance against environmental conditions present on the ISS, such as *B. pumilus* and *B. subtilis*, other *Bacillus* pose potential health risks such as *B. anthracis* [67]. Another *Bacillus* species which was isolated from the stainless steel surfaces of the ISS Arrays is *B. cereus*, an opportunistic pathogen and the causative agent of many gastrointestinal infections [68]. 

Additionally, *M. luteus* and *S. epidermidis*, which are skin commensals [69, 70], were found on the surfaces of school Touch Arrays. *S. haemolyticus*, which is also a common skin bacteria and an emerging threat in clinical isolates, was isolated from one of the polished brass surfaces [71]. Some investigations have found that *S. haemolyticus* can aid other staphylococci such as *S. aureus* to acquire antibiotic resistance genes by providing a reservoir of antibiotic resistance genes [72]. As microbial resistance towards antibiotics is an emerging threat and has been referred to as silent pandemic by the World Health Organization, interest in the use of antimicrobial surfaces has increased. Copper surfaces have been shown to be antimicrobial against a range of pathogens including MRSA [28, 73]. While copper-based surfaces have reduced microbial load when used as frequent touch surfaces in hospital settings and have shown potential in preventing healthcare-associated infections, the overall impact of copper surfaces remains controversial due to copper’s increased potential to drive development or selection of antibiotic resistance, as copper resistance has been associated with increased antibiotic resistance [26, 30, 74].

Hence, understanding the underlying mechanisms and their interaction is of pivotal importance. Long-term copper contamination in agricultural soils and copper as macro-supplement in feed can alter diversity, abundance, and mobility potential of antibiotic resistance genes, which can potentially lead to dissemination of these genes [75–77]. The presence of copper and zinc impacts antibiotic activity by binding to classes of antibiotics (e.g., ß-lactams), which drives development of antibiotic resistance in metal-exposed bacteria and in vivo efficacy [78]. Sub-lethal exposure to copper can induce oxidative stress, leading to increase in bacterial resistance to antibiotics and antibiotic and copper resistance genes co-occurred in metagenomic analysis [76, 79, 80]. For example, in MRSA, copper stress altered metabolism, induced global stress responses and copper resistance improved fitness of MRSA in infections [81–83]. Another drawback of using copper is its tendency to react with moisture and oxygen, which can potentially reduce its antibacterial efficacy as corrosion products coat the active surface [32]. Therefore, understanding the effects of frequent touching of copper surfaces and its antimicrobial efficacy is crucial.

To assess antibacterial efficacy after frequent touching, wet contact killing assays were performed using an MRSA isolate that was isolated as part of the citizen science experiment at the German schools. While other studies have found that copper-alloys reduce the microbial load on surfaces in hospitals [84], we found that copper ion release and antibacterial efficacy were significantly reduced on touched copper-containing surfaces. We have shown that while frequent touching decreases antibacterial efficiency against MRSA after 10 min exposure, untouched copper-based surfaces were antibacterial against MRSA.

Previous studies investigating antibacterial properties of surface functionalization of copper-based surfaces have shown that surface functionalization significantly increased antibacterial properties [41]. This was positively correlated with the copper ion release of the respective surface [41]. While investigation of survival of model organisms after surface exposure is essential for the development of antimicrobial surfaces, so far only new, untouched surfaces have been tested for their antibacterial efficacy, hereby disregarding organic debris which will inevitably appear during this type of application. Using contact killing assays to test antibacterial potential after frequent touching, takes deposition of putative organic contamination into account and aids in assessing antibacterial efficacy as close as possible to real life scenarios.

Reduced antibacterial efficacy and copper ion release on copper surfaces may be due to a passivation layer formed of copper oxides as well as organic contaminants such as deposits from the skin, dust, or microbiological contamination. SEM imaging of the surfaces showed that despite the intactness of the surface structures, organic debris built up around the structures. The swabbing of the surfaces for sampling could be compared to manual cleaning, but this was not sufficient to reduce the organic debris and restore full antibacterial capacity of the surfaces. However, the use of appropriate cleaning of surfaces could improve surface durability to maintain antibacterial efficacy. Therefore, exposure and touching of surfaces significantly reduced the antibacterial efficacy of most antibacterial surface types. Consequently, the application of structured surfaces for everyday use and as frequent touch surfaces, needs to be further investigated and improved, for example by using specific cleaning agents. Moreover, the 800 nm structured copper-based surfaces might be applicable for use as high-touch surfaces as the attachment of bacteria seemed to be reduced as we did not isolate any bacteria from 800 nm structured copper or brass surfaces from the ISS Touch Arrays. An ultimate goal would be to design a material which mechanically repels microorganisms from attaching to the respective material, combined with antimicrobial properties, evolution of co-resistances against the respective antibacterial agent could be put to a minimum while still reducing microbial load on high touch surfaces.

The potential of other antimicrobial surfaces has been reviewed in detail by Birkett et al. [85], and while other chemical contact-killing surfaces have antimicrobial properties such as silver, coatings etc., antimicrobial agents are always stressors which in turn lead to selective pressure towards resistance. Environmental stress is one major cause which drives development of antibiotic resistance, and one common environmental stressor is the presence of metallic ions such as copper or zinc. Additionally, previous studies have shown an increased metal resistance found in microorganisms on the ISS [9]. This would raise the question whether application of metal based antimicrobial surfaces in an environment with increased metal resistance would only amplify the selection pressure towards metal resistance. Increased confinement has been associated with loss of microbial diversity [86]. While confinement and increased cleaning have also been associated with a shift of the microbial composition from gram-positive bacteria towards gram-negative bacteria [86], we did not find a statistically significant increase in gram-positive bacteria when comparing surfaces of Touch Arrays from the ISS to Touch Arrays from schools.

In conclusion, before applying antibacterial surfaces in confined habitats and in spaceflight, their antibacterial potential must be considered for frequent use, as well as their potential to drive microbial resistance. With the help of citizen science, we were able to not only test the surfaces for spaceflight, but also for applications on Earth. Touch Arrays provided an easy-to-implement solution to test antimicrobial surfaces under real-spaceflight application, allowing us to examine the surface composition and its bacterial community as well as testing the antibacterial efficacy after direct application. Consequently, the use of Touch Arrays represents an optimal methodology for the evaluation of antimicrobial surfaces for future applications.

## Supplementary Information

Additional file 1

Additional file 2

## Data Availability

All datasets supporting the conclusions of this article are included within the article and its additional files, and can be found in a github repository ([https://github.com/jlab/microbiome_kraemer_touchingsurfaces](https:/github.com/jlab/microbiome_kraemer_touchingsurfaces)).All sequencing data is available at Qiita with the study ID 15175 as well as the European Nucleotide Archive (ERP167919) as trimmed per sample fastq files. The JupyterNotebook used for analysis can be found in the github repository: [https://github.com/jlab/microbiome_kraemer_touchingsurfaces](https:/github.com/jlab/microbiome_kraemer_touchingsurfaces).

## References

[1] García A, Lamb A, Sleptsov A, Moreno C, Victorova M, Glazkova N, et al. Post-ISS plans: what should be done? REACH. 2016;1:63–73.

[2] Santomartino R, Averesch NJ, Bhuiyan M, Cockell CS, Colangelo J, Gumulya Y, et al. Toward sustainable space exploration: a roadmap for Harnessing the power of microorganisms. Nat Commun. 2023;14(1):1391. 36944638 10.1038/s41467-023-37070-2 PMC10030976

[3] Kelley ST, Gilbert JA. Studying the microbiology of the indoor environment. Genome Biol. 2013;14(2):1–9. 10.1186/gb-2013-14-2-202 PMC3663111 23514020

[4] Peimbert M, Alcaraz LD. Where environmental Microbiome Meets its host: subway and passenger Microbiome relationships. Mol Ecol. 2023;32(10):2602–18. 35318755 10.1111/mec.16440

[5] Mora M, Mahnert A, Koskinen K, Pausan MR, Oberauner-Wappis L, Krause R, et al. Microorganisms in confined habitats: microbial monitoring and control of intensive care units, operating rooms, cleanrooms and the international space station. Front Microbiol. 2016;7:1573. 27790191 10.3389/fmicb.2016.01573 PMC5061736

[6] Mora M, Perras A, Alekhova TA, Wink L, Krause R, Aleksandrova A, et al. Resilient microorganisms in dust samples of the international space Station—survival of the adaptation specialists. Microbiome. 2016;4:1–21. 27998314 10.1186/s40168-016-0217-7 PMC5175303

[7] Byrd AL, Belkaid Y, Segre JA. The human skin Microbiome. Nat Rev Microbiol. 2018;16(3):143–55. 29332945 10.1038/nrmicro.2017.157

[8] Avila-Herrera A, Thissen J, Urbaniak C, Be NA, Smith DJ, Karouia F, et al. Crewmember Microbiome May influence microbial composition of ISS habitable surfaces. PLoS ONE. 2020;15(4):e0231838. 32348348 10.1371/journal.pone.0231838 PMC7190111

[9] Mora M, Wink L, Kögler I, Mahnert A, Rettberg P, Schwendner P, et al. Space station conditions are selective but do not alter microbial characteristics relevant to human health. Nat Commun. 2019;10(1):3990. 31488812 10.1038/s41467-019-11682-z PMC6728350

[10] Yamaguchi N, Ichijo T, Nasu M. Bacterial monitoring in the international space Station-Kibo based on rRNA gene sequence. Transactions of the Japan society for aeronautical and space sciences. Aerosp Technol Japan. 2016;14(ists30):Pp1–4.

[11] Lang JM, Coil DA, Neches RY, Brown WE, Cavalier D, Severance M, et al. A microbial survey of the international space station (ISS). PeerJ. 2017;5:e4029. 29492330 10.7717/peerj.4029 PMC5827671

[12] Singh NK, Wood JM, Karouia F, Venkateswaran K. Succession and persistence of microbial communities and antimicrobial resistance genes associated with international space station environmental surfaces. Microbiome. 2018;6(1):1–23. 30424821 10.1186/s40168-018-0585-2 PMC6234677

[13] Singh NK, Bezdan D, Checinska Sielaff A, Wheeler K, Mason CE, Venkateswaran K. Multi-drug resistant Enterobacter bugandensis species isolated from the international space station and comparative genomic analyses with human pathogenic strains. BMC Microbiol. 2018;18:1–13. 30466389 10.1186/s12866-018-1325-2 PMC6251167

[14] Tierney BT, Singh NK, Simpson AC, Hujer AM, Bonomo RA, Mason CE, et al. Multidrug-resistant acinetobacter Pittii is adapting to and exhibiting potential succession aboard the international space station. Microbiome. 2022;10(1):1–14. 36503581 10.1186/s40168-022-01358-0 PMC9743659

[15] Organization WH. WHO updates list of drug-resistant bacteria most threatening to human health. https://www.who.int/news/item/17-05-2024-who-updates-list-of-drug-resistant-bacteria-most-threatening-to-human-health (2024). Accessed 03.07.2024 2024.

[16] Girlich D, Ouzani S, Emeraud C, Gauthier L, Bonnin RA, Le Sache N, et al. Uncovering the novel Enterobacter cloacae complex species responsible for septic shock deaths in newborns: a cohort study. Lancet Microbe. 2021;2(10):e536–44. 35544179 10.1016/S2666-5247(21)00098-7

[17] Urbaniak C, Sielaff AC, Frey K, Allen J, Singh N, Jaing C, et al. Detection of antimicrobial resistance genes associated with the international space station environmental surfaces. Sci Rep. 2018;8(1):814. 29339831 10.1038/s41598-017-18506-4 PMC5770469

[18] Mehta S, Laudenslager M, Stowe R, Crucian B, Sams C, Pierson D. Multiple latent viruses reactivate in astronauts during space shuttle missions. Brain Behav Immun. 2014;41:210–7. 24886968 10.1016/j.bbi.2014.05.014

[19] Kim W, Tengra FK, Young Z, Shong J, Marchand N, Chan HK, et al. Spaceflight promotes biofilm formation by Pseudomonas aeruginosa. PLoS ONE. 2013;8(4):e62437. 23658630 10.1371/journal.pone.0062437 PMC3639165

[20] Wilson J, Ott C, Zu Bentrup KH, Ramamurthy R, Quick L, Porwollik S, et al. Space flight alters bacterial gene expression and virulence and reveals a role for global regulator Hfq. Proc Natl Acad Sci. 2007;104(41):16299–304. 17901201 10.1073/pnas.0707155104 PMC2042201

[21] Ilyin V. Microbiological status of cosmonauts during orbital spaceflights on Salyut and Mir orbital stations. Acta Astronaut. 2005;56(9–12):839–50. 15835023 10.1016/j.actaastro.2005.01.009

[22] Kim W, Tengra FK, Young Z, Shong J, Marchand N, Chan HK, et al. Spaceflight promotes biofilm formation by Pseudomonas aeruginosa. PLoS ONE. 2013;8(4):e62437. 10.1371/journal.pone.0062437. 23658630 10.1371/journal.pone.0062437 PMC3639165

[23] Russotto V, Cortegiani A, Raineri SM, Giarratano A. Bacterial contamination of inanimate surfaces and equipment in the intensive care unit. J Intensive Care. 2015;3(1):1–8. 26693023 10.1186/s40560-015-0120-5 PMC4676153

[24] Hospodsky D, Yamamoto N, Nazaroff W, Miller D, Gorthala S, Peccia J. Characterizing airborne fungal and bacterial concentrations and emission rates in six occupied children’s classrooms. Indoor Air. 2015;25(6):641–52. 25403276 10.1111/ina.12172

[25] Qian J, Hospodsky D, Yamamoto N, Nazaroff WW, Peccia J. Size-resolved emission rates of airborne bacteria and fungi in an occupied classroom. Indoor Air. 2012;22(4):339–51. 22257156 10.1111/j.1600-0668.2012.00769.x PMC3437488

[26] Weber DJ, Anderson D, Rutala WA. The role of the surface environment in healthcare-associated infections. Curr Opin Infect Dis. 2013;26(4):338–44. 23743816 10.1097/QCO.0b013e3283630f04

[27] Tiller JC. Antimicrobial surfaces. In: Börner HG, Lutz JF, editors. Bioactive Surfaces. Springer; 20 10. p.193–217.

[28] Grass G, Rensing C, Solioz M. Metallic copper as an antimicrobial surface. Appl Environ Microbiol. 2011;77(5):1541–7. 21193661 10.1128/AEM.02766-10 PMC3067274

[29] Schmidt MG, von Dessauer B, Benavente C, Benadof D, Cifuentes P, Elgueta A, et al. Copper surfaces are associated with significantly lower concentrations of bacteria on selected surfaces within a pediatric intensive care unit. Am J Infect Control. 2016;44(2):203–9. 26553403 10.1016/j.ajic.2015.09.008

[30] Colin M, Klingelschmitt F, Charpentier E, Josse J, Kanagaratnam L, De Champs C, et al. Copper alloy touch surfaces in healthcare facilities: an effective solution to prevent bacterial spreading. Materials. 2018;11(12):2479. 30563265 10.3390/ma11122479 PMC6317222

[31] Monge M, Abdel-Hady A, Aslett L, Calfee M, Williams B, Ratliff K, et al. Inactivation of MS2 bacteriophage on copper film deployed in high touch areas of a public transport system. Lett Appl Microbiol. 2022;74(3):405–10. 34862976 10.1111/lam.13624 PMC8935140

[32] Hans M, Erbe A, Mathews S, Chen Y, Solioz M, Mücklich F. Role of copper oxides in contact killing of bacteria. Langmuir. 2013;29(52):16160–6. 24344971 10.1021/la404091z

[33] Mathews S, Hans M, Mücklich F, Solioz M. Contact killing of bacteria on copper is suppressed if bacterial-metal contact is prevented and is induced on iron by copper ions. Appl Environ Microbiol. 2013;79(8):2605–11. 23396344 10.1128/AEM.03608-12 PMC3623184

[34] Elguindi J, Moffitt S, Hasman H, Andrade C, Raghavan S, Rensing C. Metallic copper corrosion rates, moisture content, and growth medium influence survival of copper ion-resistant bacteria. Appl Microbiol Biotechnol. 2011;89:1963–70. 21085951 10.1007/s00253-010-2980-x PMC3991429

[35] Hans M, Mathews S, Mücklich F, Solioz M. Physicochemical properties of copper important for its antibacterial activity and development of a unified model. Biointerphases. 2016;11(1);018902 . 10.1116/1.4935853 26577181

[36] Müller DW, Pauly C, Brix K, Kautenburger R, Mücklich F. Modifying the antibacterial performance of Cu surfaces by topographic patterning in the micro-and nanometer scale. Biomaterials Adv. 2025;169:214184. 10.1016/j.bioadv.2025.214184 39813739

[37] Siems K, Müller DW, Maertens L, Ahmed A, Van Houdt R, Mancinelli RL, et al. Testing laser-structured antimicrobial surfaces under space conditions: the design of the ISS experiment BIOFILMS. Front Space Technol. 2022;2:773244.

[38] Krämer CL, Siems K, Mueller DW, Leuko S, Mücklich F, Maurer M et al. Touching Surfaces: einfache Anwendung, große Auswirkung. In: Flugmedizin Reisemedizin Tropenmedizin. Thieme. 2024;117–22.

[39] Müller DW, Fox T, Grützmacher PG, Suarez S, Mücklich F. Applying ultrashort pulsed direct laser interference patterning for functional surfaces. Sci Rep. 2020;10(1):3647. 32108155 10.1038/s41598-020-60592-4 PMC7046748

[40] Müller DW, Holtsch A, Lößlein S, Pauly C, Spengler C, Grandthyll S, et al. In-depth investigation of copper surface chemistry modification by ultrashort pulsed direct laser interference patterning. Langmuir. 2020;36(45):13415–25. 33141584 10.1021/acs.langmuir.0c01625

[41] Müller DW, Lößlein S, Terriac E, Brix K, Siems K, Moeller R, et al. Increasing antibacterial efficiency of Cu surfaces by targeted surface functionalization via ultrashort pulsed direct laser interference patterning. Adv Mater Interfaces. 2021;8(5):2001656.

[42] Martin M. Cutadapt removes adapter sequences from high-throughput sequencing reads. EMBnet J. 2011;17(1):10–2.

[43] Gonzalez A, Navas-Molina JA, Kosciolek T, McDonald D, Vázquez-Baeza Y, Ackermann G, et al. Qiita: rapid, web-enabled Microbiome meta-analysis. Nat Methods. 2018;15(10):796–8. 30275573 10.1038/s41592-018-0141-9 PMC6235622

[44] Davis NM, Proctor DM, Holmes SP, Relman DA, Callahan BJ. Simple statistical identification and removal of contaminant sequences in marker-gene and metagenomics data. Microbiome. 2018;6:1–14. 30558668 10.1186/s40168-018-0605-2 PMC6298009

[45] Bolyen E, Rideout JR, Dillon MR, Bokulich NA, Abnet CC, Al-Ghalith GA, et al. Reproducible, interactive, scalable and extensible Microbiome data science using QIIME 2. Nat Biotechnol. 2019;37(8):852–7. 31341288 10.1038/s41587-019-0209-9 PMC7015180

[46] Garcia MA. Station Assembly Elements. https://www.nasa.gov/international-space-station/international-space-station-assembly-elements/ (2024). Accessed 04.07. 2024.

[47] Müller DW, Josten B, Wältermann S, Pauly C, Slawik S, Brix K, et al. Microstructure versus topography: the impact of crystallographic substrate modification during ultrashort pulsed direct laser interference patterning on the antibacterial properties of Cu. Front Mater. 2024;11:1397937.

[48] Hall A. Tranquility Module. https://www.nasa.gov/international-space-station/tranquility-module/ (2023). Accessed 04.07. 2024.

[49] Hall A. Destiny Laboratory Module. https://www.nasa.gov/international-space-station/destiny-laboratory-module/ (2023). Accessed 04.07. 2024.

[50] Alekhova T, Zakharchuk L, Tatarinova NY, Kadnikov V, Mardanov A, Ravin N, et al. Diversity of bacteria of the genus Bacillus on board of international space station. Doklady biochemistry and biophysics. 2015;465:347–50. 10.1134/S1607672915060010 26728721

[51] Dusane DH, Matkar P, Venugopalan VP, Kumar AR, Zinjarde SS. Cross-species induction of antimicrobial compounds, biosurfactants and quorum-sensing inhibitors in tropical marine epibiotic bacteria by pathogens and biofouling microorganisms. Curr Microbiol. 2011;62:974–80. 21086131 10.1007/s00284-010-9812-1

[52] Inkinen J, Mäkinen R, Keinänen-Toivola MM, Nordström K, Ahonen M. Copper as an antibacterial material in different facilities. Lett Appl Microbiol. 2017;64(1):19–26. 27718259 10.1111/lam.12680

[53] Bryce EA, Velapatino B, Donnelly-Pierce T, Khorami HA, Wong T, Dixon R, et al. Antimicrobial efficacy and durability of copper formulations over one year of hospital use. Infect Control Hosp Epidemiol. 2022;43(1):79–87. 33715655 10.1017/ice.2021.52

[54] Voronina OL, Kunda MS, Ryzhova NN, Aksenova EI, Semenov AN, Lasareva AV, et al. The variability of the order burkholderiales representatives in the healthcare units. Biomed Res Int. 2015;2015(1):680210. 26114111 10.1155/2015/680210 PMC4465658

[55] Stillson PT, Baltrus DA, Ravenscraft A. Prevalence of an insect-associated genomic region in environmentally acquired burkholderiaceae symbionts. Appl Environ Microbiol. 2022;88(9):e02502–21. 35435710 10.1128/aem.02502-21 PMC9088363

[56] Salido RA, Zhao HN, McDonald D, Mannochio-Russo H, Zuffa S, Oles RE, et al. The international space station has a unique and extreme microbial and chemical environment driven by use patterns. Cell. 2025;188(7):2022–41. e23. 40020666 10.1016/j.cell.2025.01.039 PMC12068931

[57] Maertens L, Coninx I, Claesen J, Leys N, Matroule J-Y, Van Houdt R. Copper resistance mediates long-term survival of Cupriavidus metallidurans in wet contact with metallic copper. Front Microbiol. 2020;11:1208. 32582116 10.3389/fmicb.2020.01208 PMC7284064

[58] Organization WH. WHO publishes list of bacteria for which new antibiotics are urgently needed. https://www.who.int/news/item/27-02-2017-who-publishes-list-of-bacteria-for-which-new-antibiotics-are-urgently-needed (2017). Accessed 03.07.2024 2024.

[59] Stinson LF, Keelan JA, Payne MS. Profiling bacterial communities in low biomass samples: pitfalls and considerations. Microbiol Australia. 2019;40(4):181–5.

[60] Kennedy KM, de Goffau MC, Perez-Muñoz ME, Arrieta M-C, Bäckhed F, Bork P, et al. Questioning the fetal Microbiome illustrates pitfalls of low-biomass microbial studies. Nature. 2023;613(7945):639–49. 36697862 10.1038/s41586-022-05546-8 PMC11333990

[61] Wilson IG. Inhibition and facilitation of nucleic acid amplification. Appl Environ Microbiol. 1997;63(10):3741–51. 9327537 10.1128/aem.63.10.3741-3751.1997 PMC168683

[62] Shamsi MH, Kraatz H-B. Interactions of metal ions with DNA and some applications. J Inorg Organomet Polym Mater. 2013;23:4–23.

[63] Amir A, McDonald D, Navas-Molina JA, Debelius J, Morton JT, Hyde E, et al. Correcting for microbial blooms in fecal samples during room-temperature shipping. Msystems. 2017;2(2). 10.1128/msystems.00199–16. 10.1128/mSystems.00199-16 PMC5340865 28289733

[64] Lauber CL, Zhou N, Gordon JI, Knight R, Fierer N. Effect of storage conditions on the assessment of bacterial community structure in soil and human-associated samples. FEMS Microbiol Lett. 2010;307(1):80–6. 20412303 10.1111/j.1574-6968.2010.01965.x PMC3148093

[65] Nicholson WL, Munakata N, Horneck G, Melosh HJ, Setlow P. Resistance of Bacillus endospores to extreme terrestrial and extraterrestrial environments. Microbiol Mol Biol Rev. 2000;64(3):548–72. 10974126 10.1128/mmbr.64.3.548-572.2000 PMC99004

[66] Checinska A, Paszczynski A, Burbank M. Bacillus and other spore-forming genera: variations in responses and mechanisms for survival. Annual Rev Food Sci Technol. 2015;6(1):351–69. 25705935 10.1146/annurev-food-030713-092332

[67] Quagliariello A, Cirigliano A, Rinaldi T. Bacilli in the international space station. Microorganisms. 2022;10(12):2309. 36557562 10.3390/microorganisms10122309 PMC9782108

[68] Kotiranta A, Lounatmaa K, Haapasalo M. Epidemiology and pathogenesis of Bacillus cereus infections. Microbes Infect. 2000;2(2):189–98. 10742691 10.1016/s1286-4579(00)00269-0

[69] Brown MM, Horswill AR. Staphylococcus epidermidis—Skin friend or foe? PLoS Pathog. 2020;16(11):e1009026. 33180890 10.1371/journal.ppat.1009026 PMC7660545

[70] Kloos WE, Musselwhite MS. Distribution and persistence of Staphylococcus and micrococcus species and other aerobic bacteria on human skin. Appl Microbiol. 1975;30(3):381–95. 810086 10.1128/am.30.3.381-395.1975 PMC187193

[71] Czekaj T, Ciszewski M, Szewczyk EM. Staphylococcus haemolyticus–an emerging threat in the Twilight of the antibiotics age. Microbiology. 2015;161(Pt11):2061–8. 26363644 10.1099/mic.0.000178

[72] Fluit AC, Carpaij N, Majoor EA, Bonten MJ, Willems RJ. Shared reservoir of CcrB gene sequences between coagulase-negative Staphylococci and methicillin-resistant Staphylococcus aureus. J Antimicrob Chemother. 2013;68(8):1707–13. 23599362 10.1093/jac/dkt121

[73] Steindl G, Heuberger S, Springer B. Antimicrobial effect of copper on multidrug-resistant bacteria. Vet Med Austria. 2012;99:38–43.

[74] Salgado CD, Sepkowitz KA, John JF, Cantey JR, Attaway HH, Freeman KD, et al. Copper surfaces reduce the rate of healthcare-acquired infections in the intensive care unit. Infect Control Hosp Epidemiol. 2013;34(5):479–86. 23571364 10.1086/670207

[75] Hu HW, Wang JT, Li J, Li JJ, Ma YB, Chen D, et al. Field-based evidence for copper contamination induced changes of antibiotic resistance in agricultural soils. Environ Microbiol. 2016;18(11):3896–909. 27207327 10.1111/1462-2920.13370

[76] Xu Y, Xu J, Mao D, Luo Y. Effect of the selective pressure of sub-lethal level of heavy metals on the fate and distribution of ARGs in the catchment scale. Environ Pollut. 2017;220:900–8. 27876226 10.1016/j.envpol.2016.10.074

[77] Yazdankhah S, Rudi K, Bernhoft A. Zinc and copper in animal feed–development of resistance and co-resistance to antimicrobial agents in bacteria of animal origin. Microb Ecol Health Disease. 2014;25(1):25862. 10.3402/mehd.v25.25862 PMC4179321 25317117

[78] Poole K. At the nexus of antibiotics and metals: the impact of Cu and Zn on antibiotic activity and resistance. Trends Microbiol. 2017;25(10):820–32. 28526548 10.1016/j.tim.2017.04.010

[79] Liu W, Xu Y, Slaveykova VI. Oxidative stress induced by sub-lethal exposure to copper as a mediator in development of bacterial resistance to antibiotics. Sci Total Environ. 2023;860:160516. 36470380 10.1016/j.scitotenv.2022.160516

[80] Li X, Rensing C, Vestergaard G, Arumugam M, Nesme J, Gupta S, et al. Metagenomic evidence for co-occurrence of antibiotic, biocide and metal resistance genes in pigs. Environ Int. 2022;158:106899. 34598063 10.1016/j.envint.2021.106899

[81] Baker J, Sitthisak S, Sengupta M, Johnson M, Jayaswal RK, Morrissey JA. Copper stress induces a global stress response in Staphylococcus aureus and represses Sae and Agr expression and biofilm formation. Appl Environ Microbiol. 2010;76(1):150–60. 10.1128/AEM.02268-09. 19880638 10.1128/AEM.02268-09 PMC2798663

[82] Saenkham-Huntsinger P, Hyre AN, Hanson BS, Donati GL, Adams LG, Ryan C, et al. Copper resistance promotes fitness of Methicillin-Resistant Staphylococcus aureus during urinary tract infection. mBio. 2021;12(5):e0203821. 10.1128/mBio.02038-21. 34488457 10.1128/mBio.02038-21 PMC8546587

[83] Tarrant E, G PR, McIlvin MR, Stevenson J, Barwinska-Sendra A, Stewart LJ, et al. Copper stress in Staphylococcus aureus leads to adaptive changes in central carbon metabolism. Metallomics. 2019;11(1):183–200. 10.1039/c8mt00239h. 30443649 10.1039/c8mt00239h PMC6350627

[84] Karpanen TJ, Casey A, Lambert PA, Cookson B, Nightingale P, Miruszenko L, et al. The antimicrobial efficacy of copper alloy furnishing in the clinical environment: a crossover study. Infect Control Hosp Epidemiol. 2012;33(1):3–9. 22173515 10.1086/663644

[85] Birkett M, Dover L, Cherian Lukose C, Wasy Zia A, Tambuwala MM, Serrano-Aroca A. Recent advances in Metal-Based antimicrobial coatings for High-Touch surfaces. Int J Mol Sci. 2022;23(3). 10.3390/ijms23031162. 10.3390/ijms23031162 PMC8835042 35163084

[86] Mahnert A, Moissl-Eichinger C, Zojer M, Bogumil D, Mizrahi I, Rattei T, et al. Man-made microbial resistances in built environments. Nat Commun. 2019;10(1):968. 30814504 10.1038/s41467-019-08864-0 PMC6393488

