# Supplementary material for: Touching Surfaces – Presence of microorganisms on antimicrobial metal surfaces on the International Space Station and in German schools
Source: BMC Microbiol. 2025 Oct 6;25:631. doi: 10.1186/s12866-025-04316-6 (PMC12502293; doi:10.1186/s12866-025-04316-6)
Supplement: Supplementary file 1 — Additional file 1 [file 12866_2025_4316_MOESM1_ESM.docx]

# Additional Files

## Additional Figures


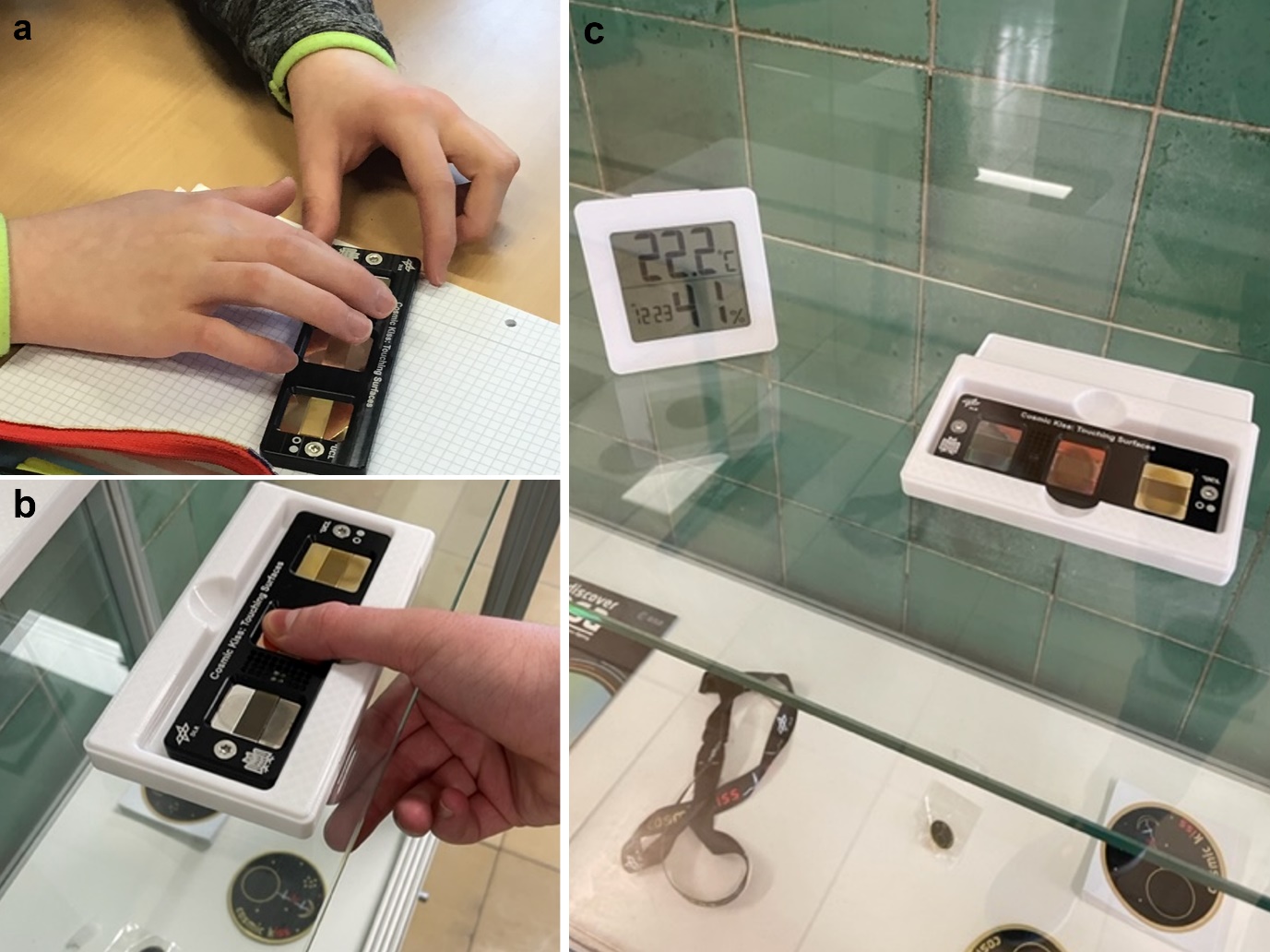


**Additional Figure 1: Exemplary Touch Events and setup of Touch Arrays in schools**. (a) and (b) show exemplary Touch Events in schools. (c) shows an exemplary setup of a Touch Array in a case in school.


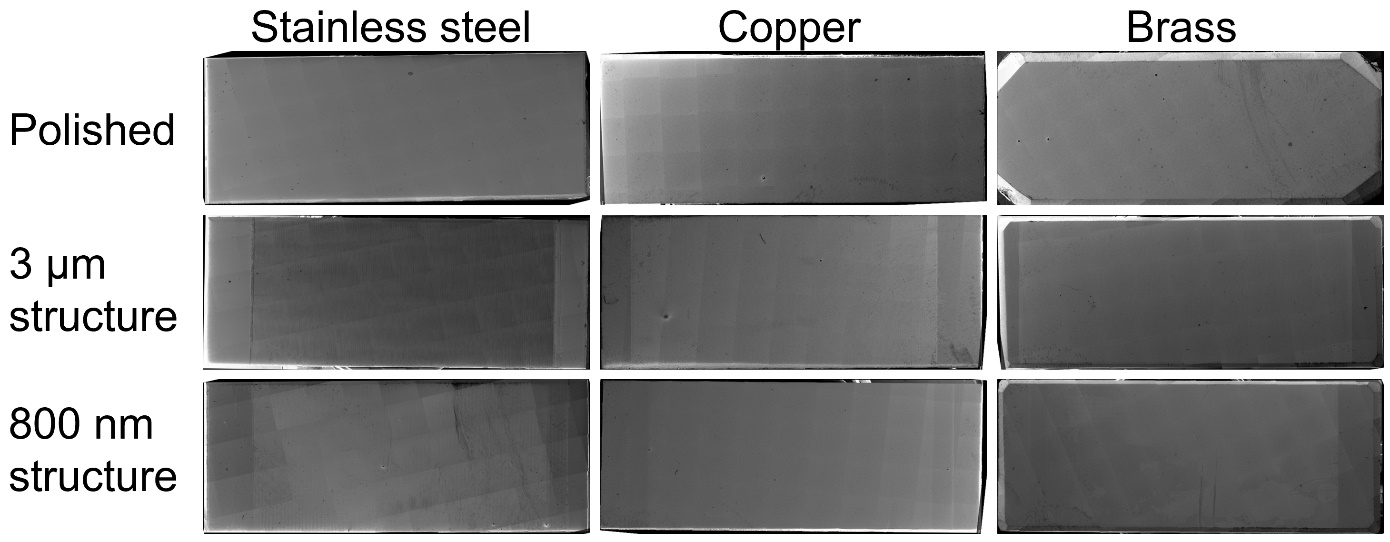


**Additional Figure 2:** **Mosaic images of surfaces from untouched surfaces as they would be assembled in the Touch Arrays.** Using SEM, a mapping of all surfaces of a Touch Array was created. The assembly of the images resembles the assembly of the surfaces in the Touch Array. The mapping shows the whole surface of the Touch Array, and each surface has the dimension of 10 mm x 25 mm respectively.


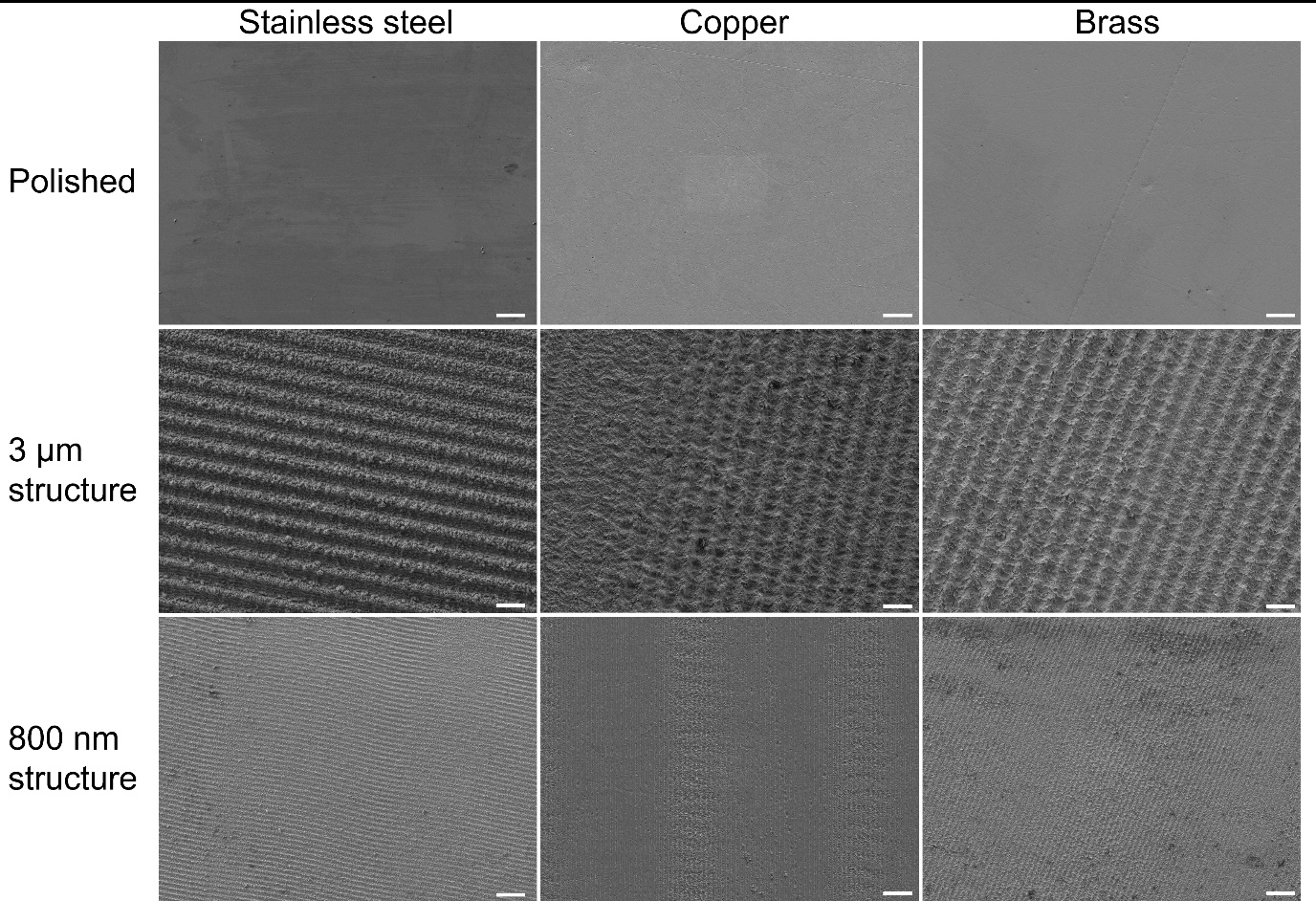


**Additional Figure 3: SEM images of untouched sample plates** (scale bar = 4 µm). Untouched surfaces were analyzed using scanning electron microscopy. The upper legend indicates the respective metal, and the left legend describes the surface pattern of each surface sample. Sample surfaces were chemically fixed and imaged without previous sputtering. Pixel size of each picture is 27.91 nm.


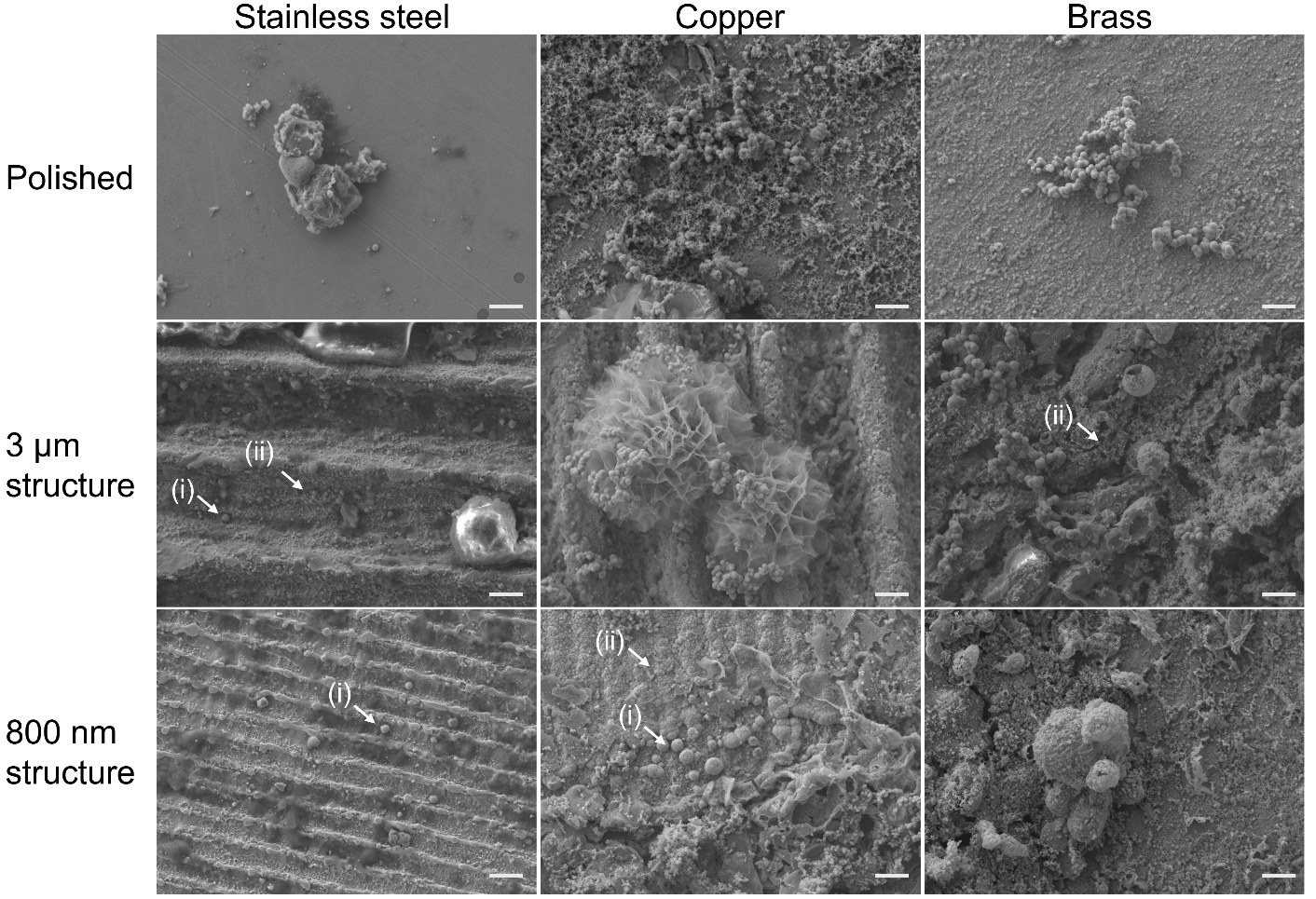


**Additional Figure 4:** **SEM images of sample plates of school Touch Array (School-E) after frequent touching** (scale bar = 1 µm). The upper legend provides the respective metal, and the left legend describes the surface pattern of each surface sample. Sample surfaces were located in a school in Germany, where they were regularly touched by students. Sample surfaces were chemically fixed and imaged without previous sputtering.


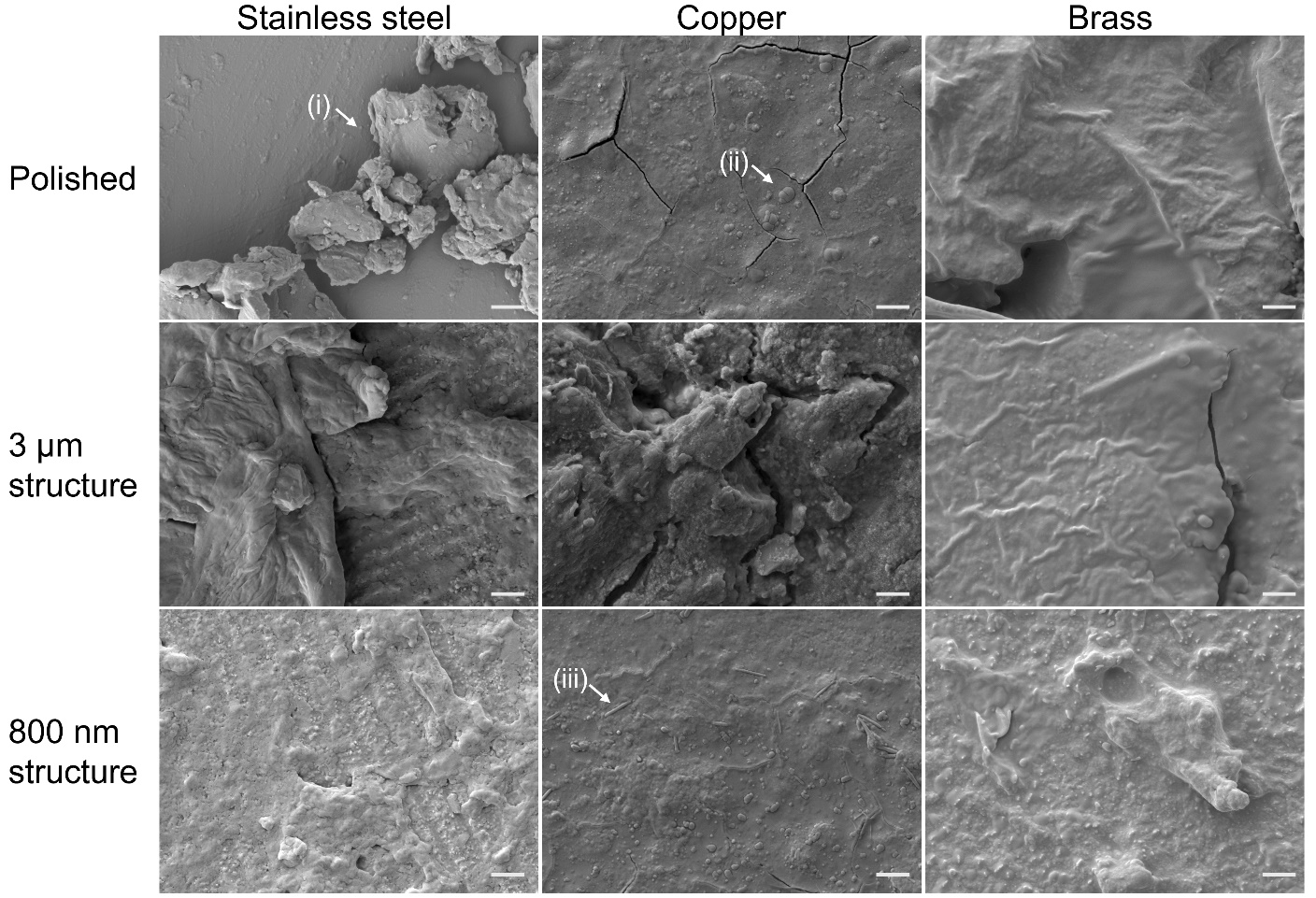


**Additional Figure 5: SEM images of sample plates of ISS Touch Array (ISS-E) after frequent touching** (scale bar = 1 µm). The upper legend provides the respective metal, and the left legend describes the surface pattern of each surface sample. The respective Touch Array was installed inside the Columbus module on the ISS, where its surfaces were regularly touched by astronauts. The surfaces of the individual plates were not chemically fixed. The 3 µm structured copper sample was already sputter-coated with silver onboard the ISS during a space experiment. For the other surfaces, half of the surface was sputter-coated with palladium.


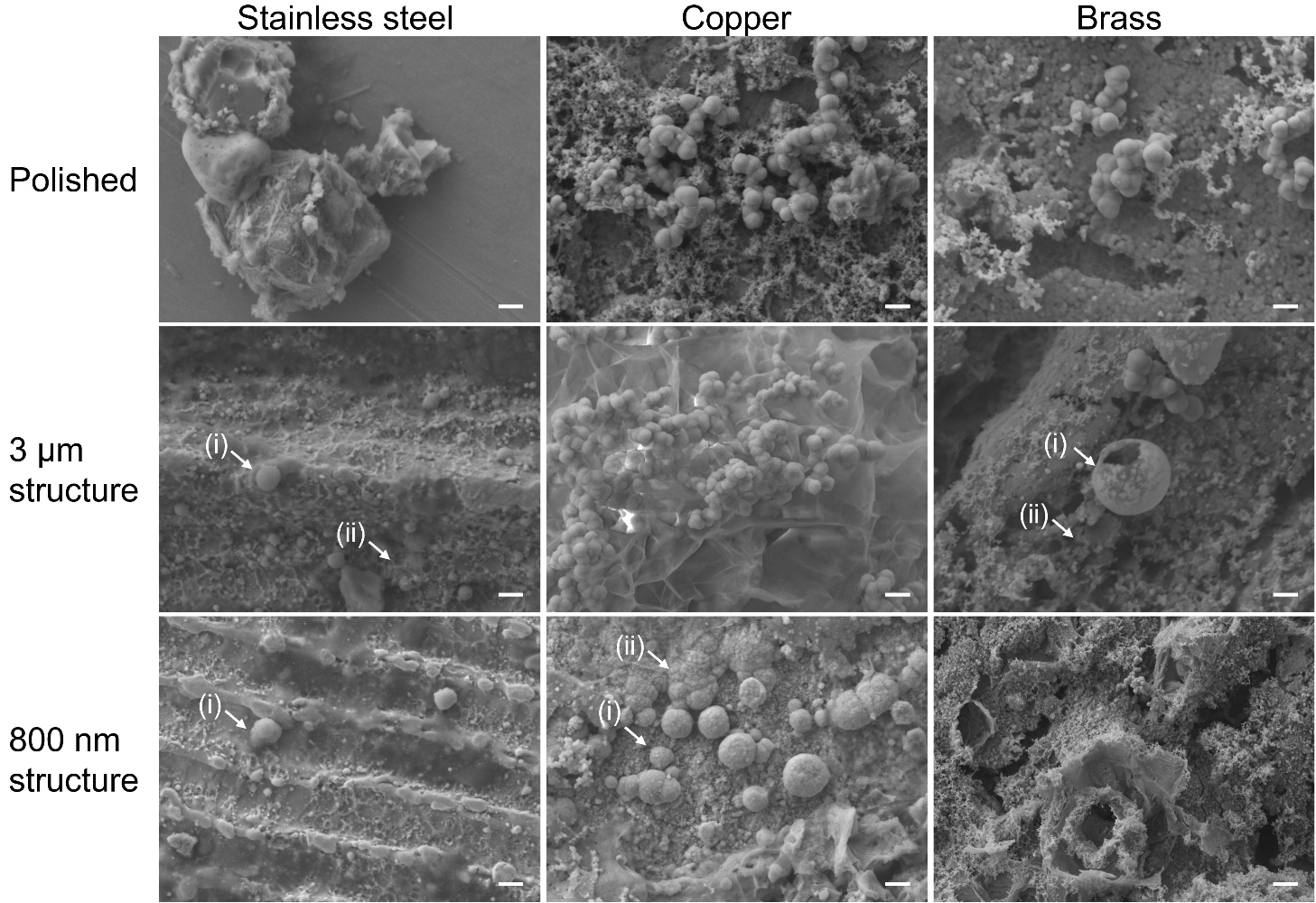


**Additional Figure 6: SEM images of sample plates of school Touch Array (School-E) after frequent touching** (scale bar = 300 nm). The upper legend provides the respective metal, and the left legend describes the surface pattern of each surface sample. Sample surfaces were located in a school in Germany, where they were regularly touched by students. Sample surfaces were chemically fixed and imaged without previous sputtering.


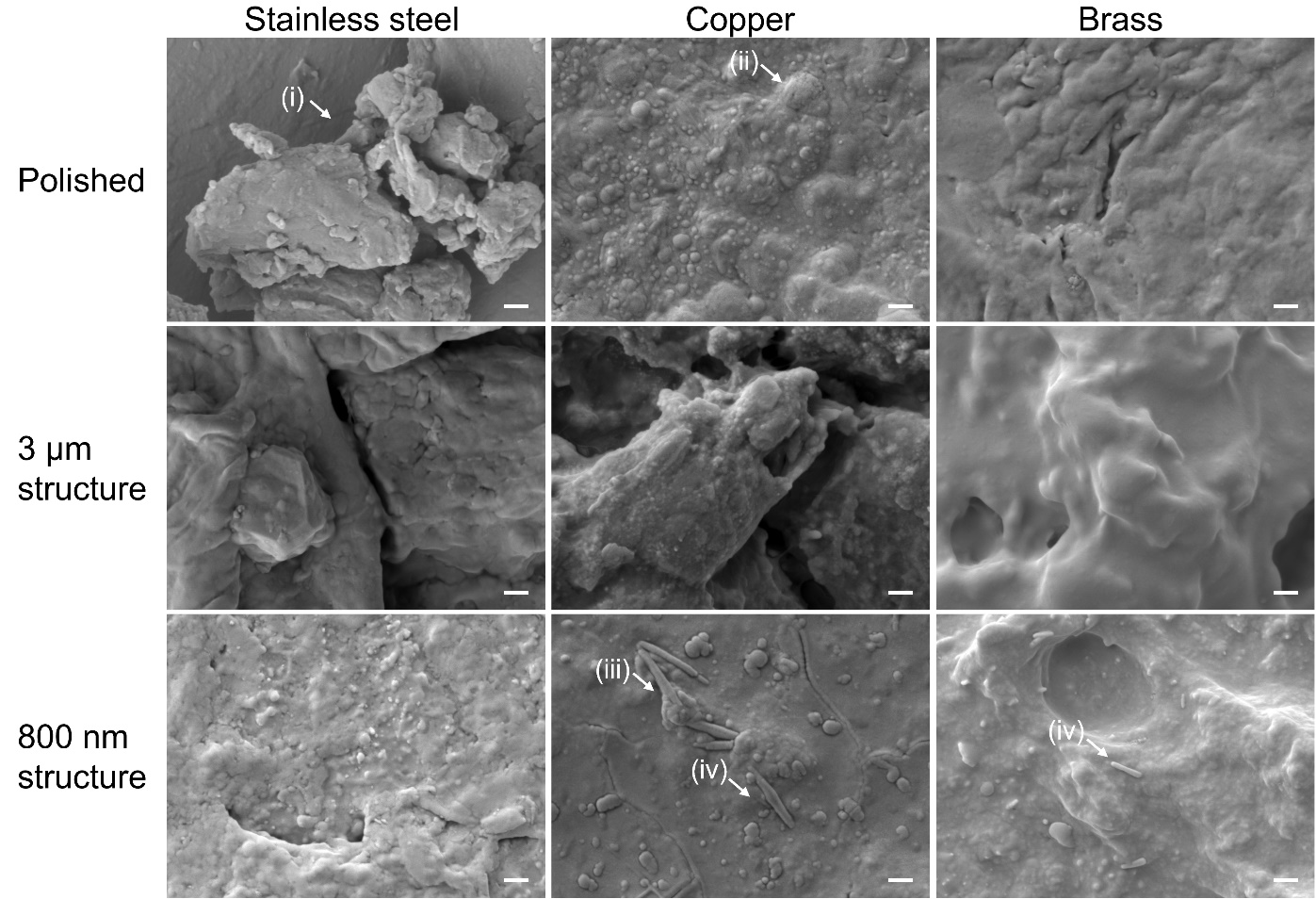


**Additional Figure 7:** **SEM images of sample plates of ISS Touch Array (ISS-E) after frequent touching** (scale bar = 300 nm). The upper legend provides the respective metal, and the left legend describes the surface pattern of each surface sample. The respective Touch Array was installed inside the Columbus module on the ISS, where its surfaces were regularly touched by astronauts. The surfaces of the individual plates were not chemically fixed. The 3 µm structured copper sample was already sputter-coated with silver onboard the ISS during a space experiment. For the other surfaces, half of the surface was sputter-coated with palladium.


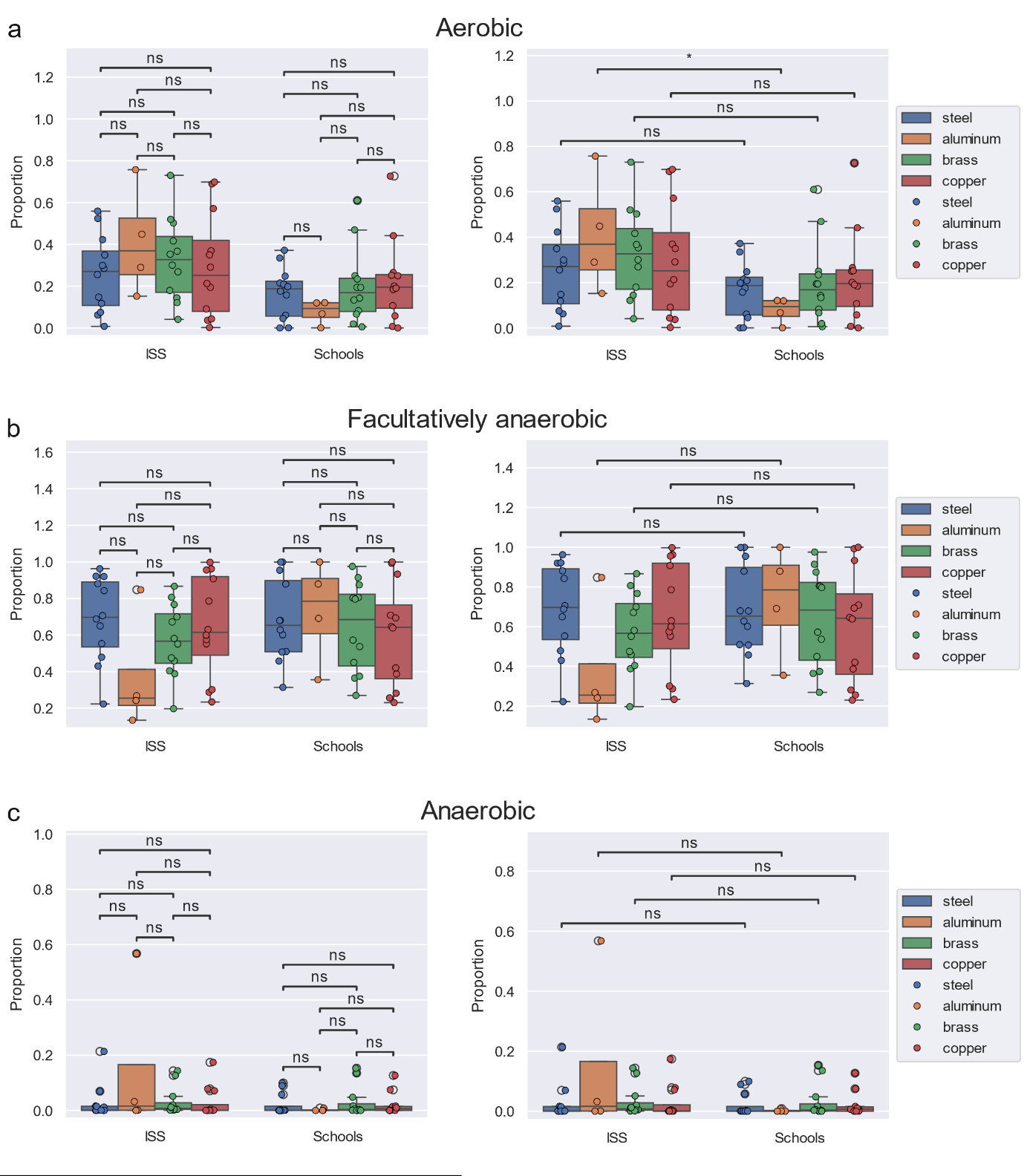


**Additional Figure 8: Abundance of aerobic, facultative anaerobic, and anaerobic bacteria on the different surfaces dependent on the surface metal and their location (ISS and schools).** Using bugbase, the NGS dataset was explored for statistically significant abundance of different traits of bacteria.


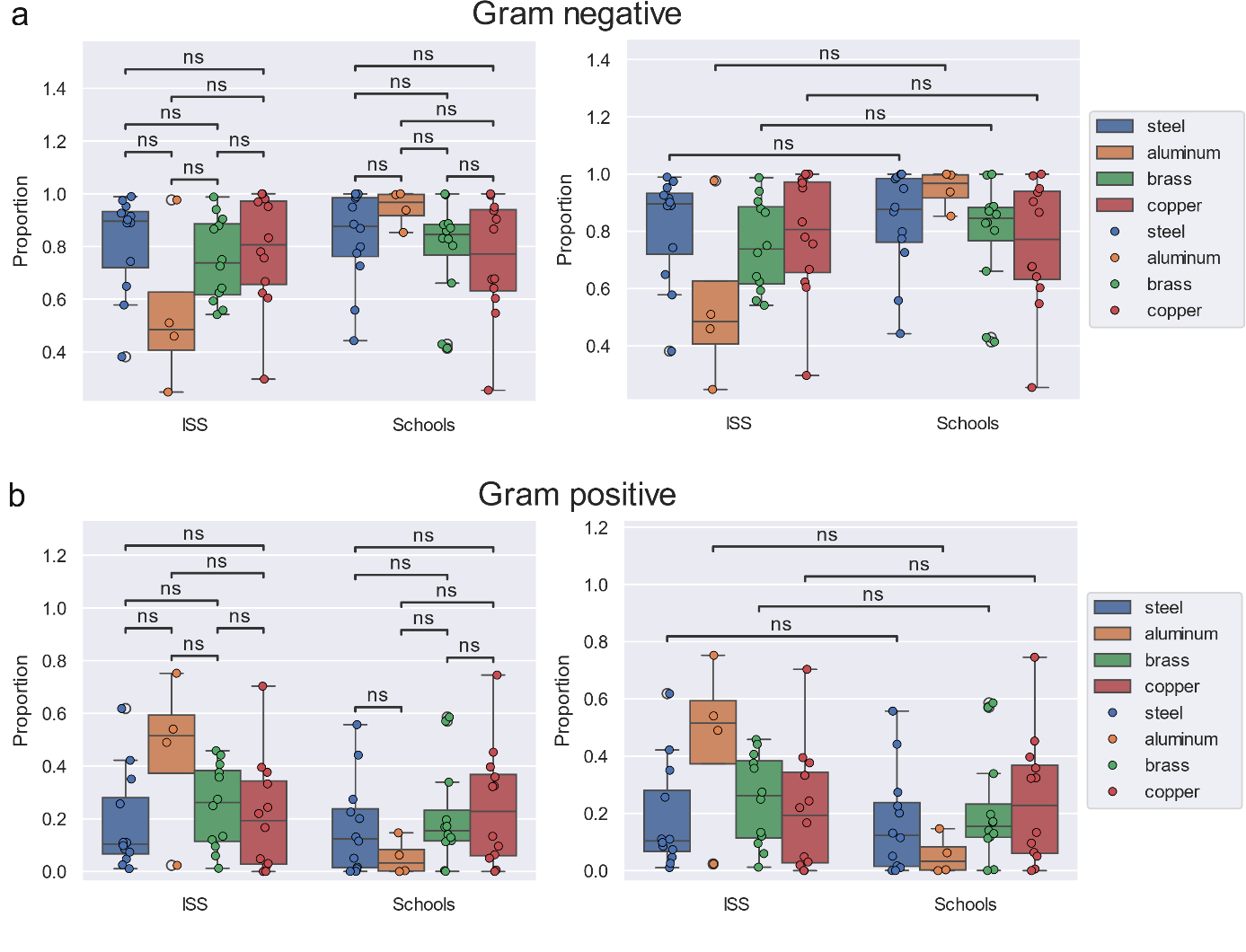


**Additional Figure 9: Abundance of gram-negative and gram-positive bacteria on the different surfaces dependent on the surface metal and their location (ISS and schools).** Using bugbase, the NGS dataset was explored for statistically significant abundance of different traits of bacteria.


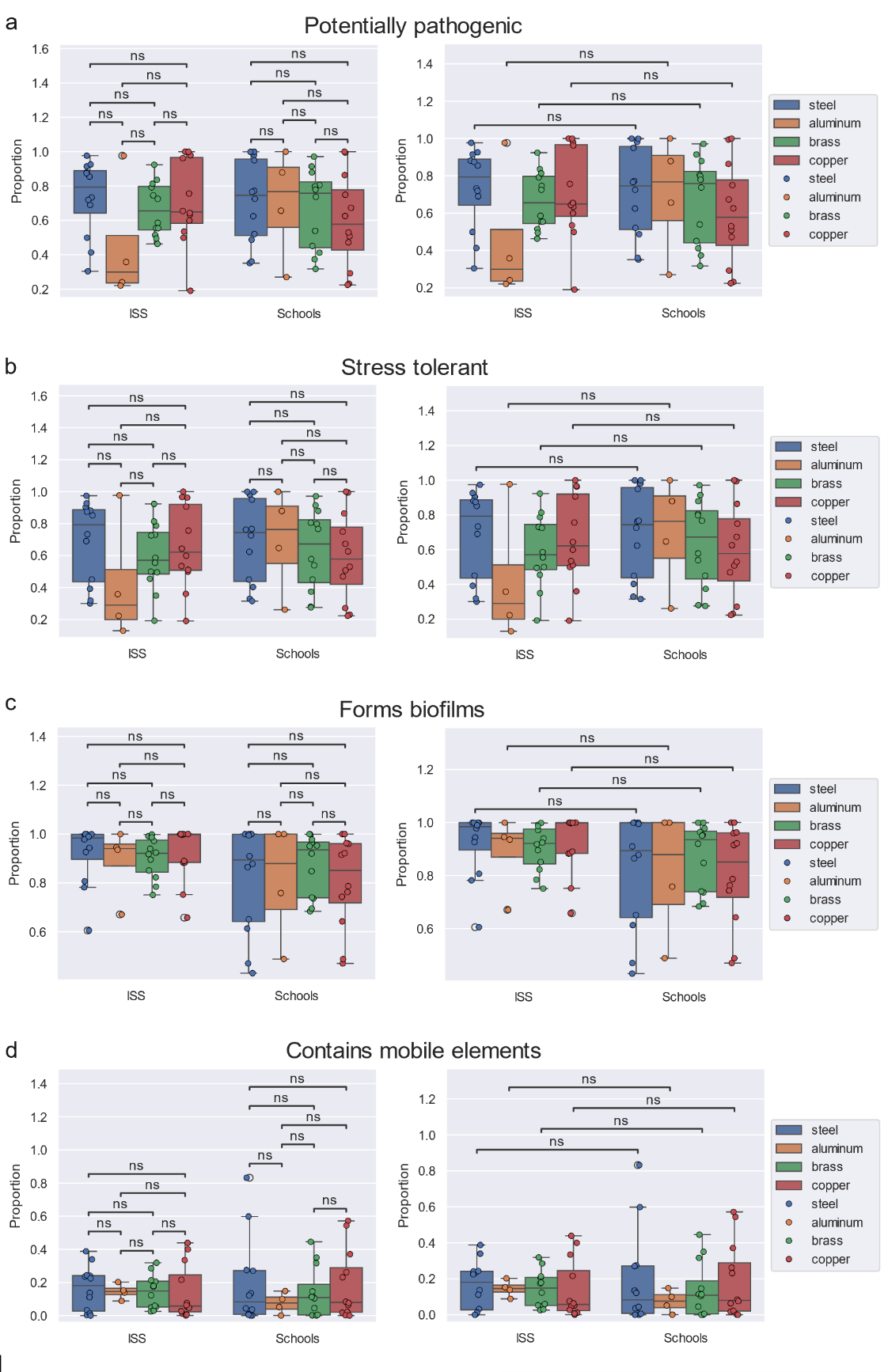


**Additional Figure 10: Abundance of potentially pathogenic (a), stress tolerant (b), biofilm forming (c), and mobile elements containing (d) bacteria in respect to the surface metal and their location (ISS and schools).** Using bugbase, the NGS dataset was explored for statistically significant abundance of different traits of bacteria.


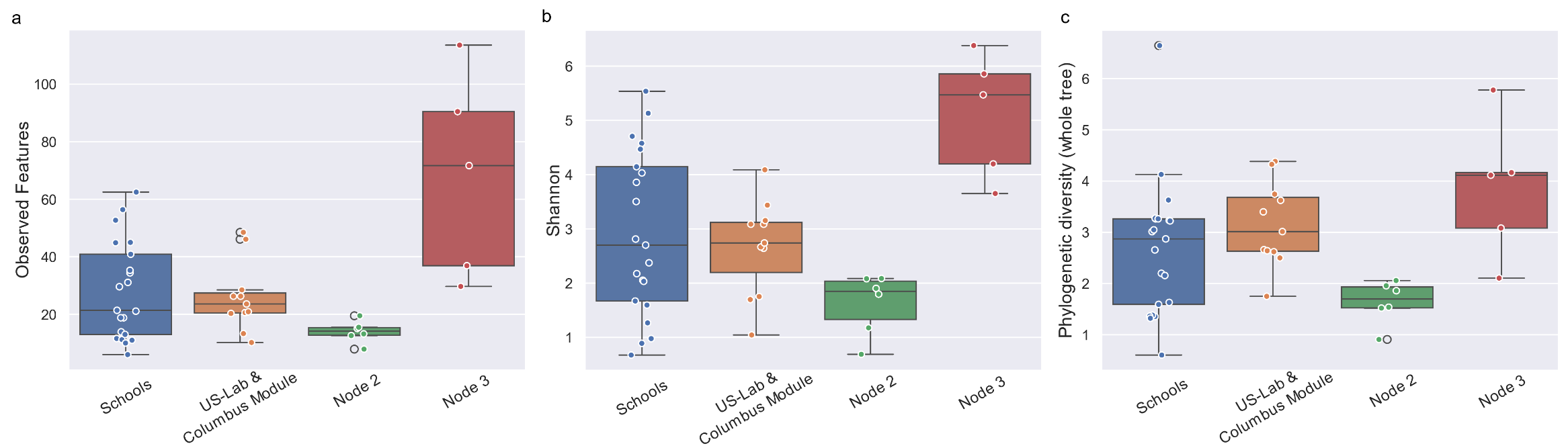


**Additional Figure 11: Diversity measures based on population of location of respective Touch Array.** Samples were grouped based on the population of the area where the Touch Array was located. The diversity measures observed features (a), Shannon index (b), and phylogenetic diversity (c) are given for the surfaces of schools, laboratory modules on the ISS (US-Lab and Columbus module), Node 2 and Node 3.


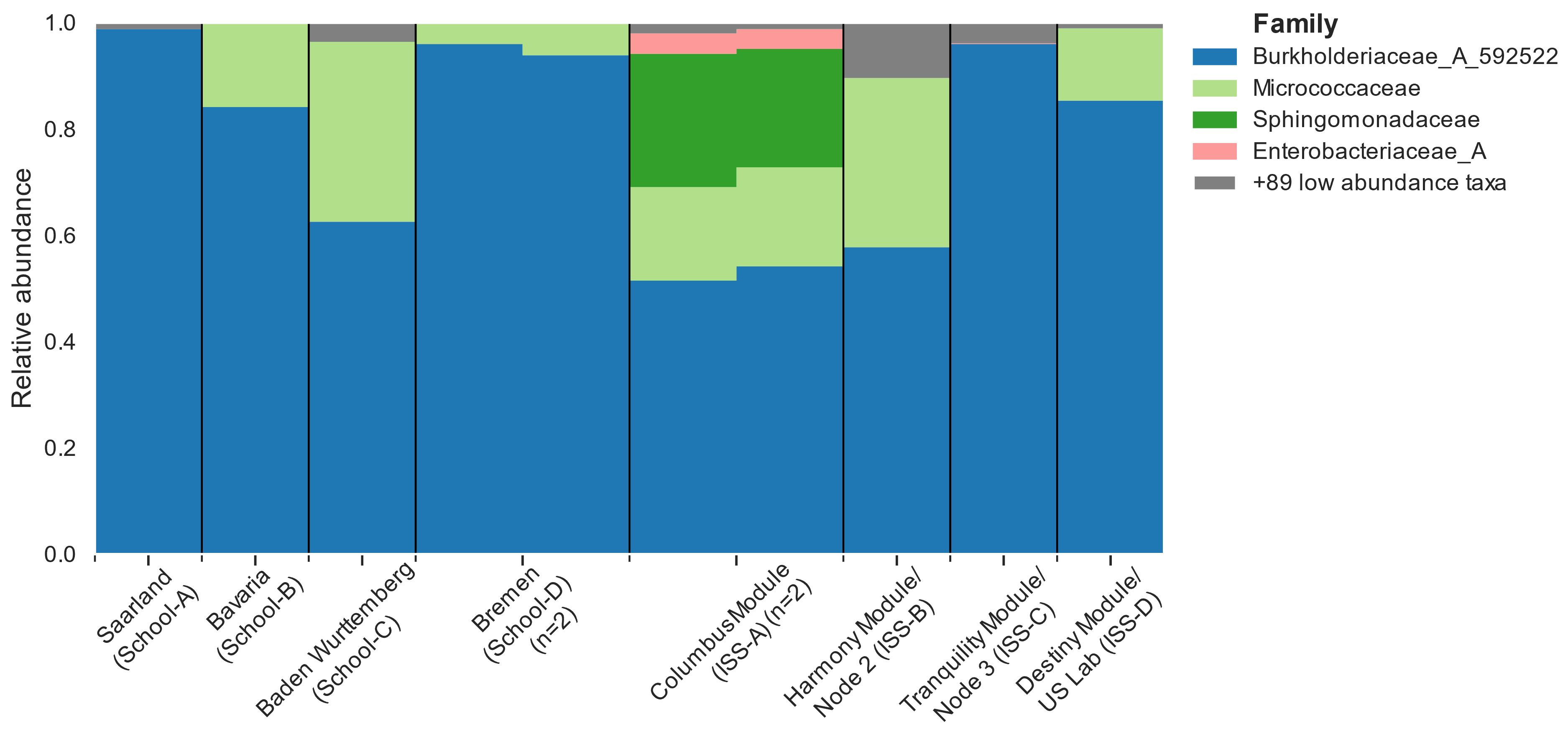


**Additional Figure 12: Relative abundance of bacteria on family level of sequenced blank swabs.** Taxa bar plot of the detected bacteria of blank swabs on family level are given grouped by their respective controls. Blank swabs were taken during swabbing of Touch Arrays (controls). Sample number per group is n=1, if not stated otherwise.

## Additional Tables:

**Additional Table 1: Environmental data during exposure of Touch Arrays and Touch Events.** Temperature and humidity were documented regularly during participation of schools in Touching Surfaces project. Environmental data was measured using a thermo-/hygrometer. (Additional Table 1-EnvData)

**Additional Table 2: Statistical significance for wet contact killing data**: CFU mL^-1^. A one-way ANOVA on ranks was performed, and when the mean difference was greater than would be expected by chance a multiple comparison procedure was performed to isolate groups which differ from the others. If the median values did not differ greater than would be expected by chance, no multiple comparison procedure was tested (Sample groups: S1, C1). G= ground/schools, ISS= International Space Station, UT= untouched; S= stainless steel, C= copper, B= brass; 1= polished, 2a=3 µm structure, 2b= 800 nm structure. (Additional Table 2-CFUmL-1)

**Additional Table 3: Statistical significance for copper concentration in MRSA wet contact killing assay**: A one-way ANOVA on ranks was performed, and when the mean difference was greater than would be expected by chance a multiple comparison procedure was performed to isolate groups which differ from the others. Median values of sample groups S2a, S2b, and C2b did not differ greater than would be expected by chance, no multiple comparison procedure was tested. G= ground/schools, ISS= International Space Station, UT= untouched; S= stainless steel, C= copper, B= brass; 1= polished, 2a=3 µm structure, 2b= 800 nm structure. (Additional Table 3-Cu-MRSA)

**Additional Table 4: Statistical significance for copper concentration of PBS after 10 min surface exposure**: A one-way ANOVA on ranks was performed, and when the mean difference was greater than would be expected by chance a multiple comparison procedure was performed to isolate groups which differ from the others. Median values of sample groups S2a and C1 did not differ greater than would be expected by chance, no multiple comparison procedure was tested. G= ground/schools, ISS= International Space Station, UT= untouched; S= stainless steel, C= copper, B= brass; 1= polished, 2a=3 µm structure, 2b= 800 nm structure. (Additional Table 4-Cu-PBS)

**Additional Table 5: Diversity measures of samples grouped according to ISS and ground/school surfaces.** For alpha diversity calculations, the metrics observed features, Shannon index, and Faith’s phylogenetic diversity index were used. Statistical significance was tested with Mann-Whitney-Wilcoxon tests. For beta diversity calculations the metric weighted and unweighted UniFrac and Bray Curtis were used. Statistical significance was tested using PERMANOVA tests with 999 permutations. The total number of performed tests is n=3, p-values which are smaller than the FDR corrected alpha value are marked in red (p ≤ 0.016666667) Sample group GER:NRW are blank swabs, which were taken as a control during swabbing of Touch Arrays (Additional Table 5-ISS & Sch).

**Additional Table 6: Diversity measures of bacterial composition on Touch Array surfaces grouped dependent on their location on the ISS and in schools.** For alpha diversity calculations, the metrics observed features, Shannon index, and Faith’s phylogenetic diversity index were used. Statistical significance was tested with Mann-Whitney-Wilcoxon tests. For beta diversity calculations the metric weighted and unweighted UniFrac and Bray Curtis were used. Statistical significance was tested using PERMANOVA tests with 999 permutations. The total number of performed tests is n=28, p-values which are smaller than the FDR corrected alpha value are marked in red (p ≤ 0.001785714). (Additional Table 6-Location)

**Additional Table 7: Diversity measures of bacterial composition on surfaces grouped dependent on the experiment type (ISS/schools) and blank swab controls.** For alpha diversity calculations, the metrics observed features, Shannon index, and Faith’s phylogenetic diversity index were used. Statistical significance was tested with Mann-Whitney-Wilcoxon tests. For beta diversity calculations the metric weighted and unweighted UniFrac and Bray Curtis were used. Statistical significance was tested using PERMANOVA tests with 999 permutations. The total number of performed tests is n=6, p-values which are smaller than the FDR corrected alpha value are marked in red (p ≤ 0.008333333). (Additional-Table 7-wControl)

**Additional Table 8: Diversity measures of bacterial composition on surfaces grouped by surface types (metal and topography).** For alpha diversity calculations, the metrics observed features, Shannon index, and Faith’s phylogenetic diversity index were used. Statistical significance was tested with Mann-Whitney-Wilcoxon tests. For beta diversity calculations the metric weighted and unweighted UniFrac and Bray Curtis were used. Statistical significance was tested using PERMANOVA tests with 999 permutations. The total number of performed tests is n=6, p-values which are smaller than the FDR corrected alpha value are marked in red (p ≤ 0.008333333). (Additional Table 8-Surf type)

**Additional Table 9: Diversity measures of bacterial communities grouped by surface topography.** For alpha diversity calculations, the metrics observed features, Shannon index, and Faith’s phylogenetic diversity index were used. Statistical significance was tested with Mann-Whitney-Wilcoxon tests. For beta diversity calculations the metric weighted and unweighted UniFrac and Bray Curtis were used. Statistical significance was tested using PERMANOVA tests with 999 permutations. The total number of performed tests is n=10, p-values which are smaller than the FDR corrected alpha value are marked in red (p ≤ 0.005). (Additional Table9-Topography)

**Additional Table 10. Diversity measures of bacterial composition on surfaces dependent on the metal of the surface.** For alpha diversity calculations, the metrics observed features, Shannon index, and Faith’s phylogenetic diversity index were used. Statistical significance was tested with Mann-Whitney-Wilcoxon tests. For beta diversity calculations the metric weighted and unweighted UniFrac and Bray Curtis were used. Statistical significance was tested using PERMANOVA tests with 999 permutations. The total number of performed tests is n=10, p-values which are smaller than the FDR corrected alpha value are marked in red (p ≤ 0.005). (Additional Table 10-Metal)

**Additional Table 11. Diversity measures of bacterial composition on surfaces dependent on their location in populated and less populated areas.** For alpha diversity calculations, the metrics observed features, Shannon index, and Faith’s phylogenetic diversity index were used. Statistical significance was tested with Mann-Whitney-Wilcoxon tests. For beta diversity calculations the metric weighted and unweighted UniFrac and Bray Curtis were used. Statistical significance was tested using PERMANOVA tests with 999 permutations. The total number of performed tests is n=6, p-values which are smaller than the FDR corrected alpha value are marked in red (p ≤ 0.008333333). (Additional Table 11-PopAreas)
